# Pro-inflammatory macrophages suppress HIV replication in humanized mice and *ex vivo* co-cultures

**DOI:** 10.3389/fimmu.2024.1439328

**Published:** 2024-11-07

**Authors:** Luca Baroncini, Christina K. S. Muller, Nicole P. Kadzioch, Rebekka Wolfensberger, Doris Russenberger, Simon Bredl, Tafadzwa Mlambo, Roberto F. Speck

**Affiliations:** Department of Infectious Diseases and Hospital Epidemiology, University of Zurich, University Hospital of Zurich, Zurich, Switzerland

**Keywords:** HIV-1, macrophages, iHMD-NSG mice, humanized mice for HIV, myeloid cells

## Abstract

**Introduction:**

Very little is known about the role of macrophages as immune mediators during natural HIV infection. Humanized mice are an extremely valuable *in vivo* model for studying HIV pathogenesis. However, the presence of murine mononuclear phagocytes in these models represents a significant limitation for studying their human counterpart. Therefore, we have developed a novel humanized mouse model that allows selective depletion of human myeloid cells at a time point of our choosing.

**Methods:**

We genetically engineered human hematopoietic stem and progenitor cells (HSPCs) to express an inducible caspase-9 (iCas9) suicide system under a synthetic myeloid promoter. Using these HSPCs, we generated humanized mice. iCasp9 induction *in vivo* resulted in selective human myeloid cell death in this inducible human myeloid depletion (iHMD) mouse model. In addition, we co-cultured monocyte-derived macrophages with *ex vivo* HIV-infected PBMCs to further mechanistically investigate the effect of macrophages on HIV replication using flow cytometry, cytokine analysis, and RNA sequencing of both macrophages and CD4+ T cells.

**Results:**

HIV infection induced a pro-inflammatory phenotype in HIV-infected humanized NSG mice during the early and late stages of HIV infection. Myeloid cell depletion in HIV-infected iHMD-NSG mice resulted in a rapid increase in HIV RNA replication, which was accompanied by a loss of pro-inflammatory cytokines. Co-culture of macrophages with *ex vivo* HIV-infected PBMCs reproduced their anti-HIV effects observed *in vivo*. Transcriptomic data showed macrophages upregulate antiviral cytokines and chemokines in co-culture, while inducing CD4+ T cells to upregulate HIV restriction factors and downregulate pathways involved in protein expression and cell replication.

**Discussion:**

This study describes a novel role of macrophages as effector cells, both *ex vivo* and *in vivo*, acting against HIV replication and limiting disease progression.

## 1 Introduction

Macrophages play a crucial role in the innate immune response as well as in maintaining tissue homeostasis and facilitating repair (1). These vastly different tasks require a tremendous plasticity orchestrated by various receptors responding to their cognate ligands, which eventually directs the macrophage differentiation (2). Macrophages are distributed virtually in every organ, where they act as the first line of defense against pathogens, among which viruses (3, 4). Macrophage numbers increase during the early stages of viral infections, including hepatitis C virus (HCV), hepatitis B (HBV) and SARS-CoV2 infection (5–7). These events are usually followed by an upregulation of pro-inflammatory cytokines, resulting in the recruitment of effector cells to the infected tissue and antigen presentation to T cells (8). Macrophages themselves play a central role by killing infected cells and restricting viral infection (9). However, different viruses alter macrophages’ phenotype and functionality, leading to aberrant responses, including immunopathology. For example, macrophages contribute to the insurgency of liver fibrosis during HCV and HBV infection via TGF-1β, IL-1β and TNFα production (5, 6). The alteration in number and functionality of alveolar macrophages are associated with cytokine storm and severe disease prognosis during SARS-CoV-2 infection (10).

In human immunodeficiency virus (HIV) type-1 infection, macrophages have long been viewed as just another HIV-permissive cell that functions as an HIV producer but rarely experiences viral cytopathicity (11). Notably, macrophages can be infected via direct membrane fusion or upon engulfment of HIV-infected CD4+ T cells (12). Furthermore, HIV can hijack macrophages by accumulating in virus-containing compartments (VCCs). These VCCs release HIV-free virions when interacting with activated CD4+ T cells (13). As the HIV infection progresses, HIV quasi-species within the host become increasingly more macrophage-tropic (14). The ability of HIV strains to replicate in macrophages in the absence of T cells was corroborated by *in vivo* studies in non-human primates and the “macrophage-only humanized mouse model” (15, 16).

Macrophages, such as those found in mucosal tissue, are now considered key cells in virus transmission during sexual contact and then in virus dissemination (17). Depending on the tissue origin, macrophages have a variable half-life ranging from just a few weeks to over four years in the brain (18, 19). Together with their self-renewal capacity (20), this suggests an involvement of these cells in the formation of the HIV reservoir. In fact, latently HIV-infected macrophages are now considered a significant part of the HIV reservoir in people living with HIV and must be targeted to achieve control without combined antiretroviral therapy (cART).

But what about macrophages’ anti-HIV activity? The cells with well-documented anti-HIV activity are cytotoxic CD8+ T cells and natural killer (NK) cells (21, 22). Macrophages’ sensing of HIV during the early phases of infection triggers an inflammatory cascade, polarizing macrophages toward a pro-inflammatory state (23, 24). As HIV infection progresses toward the chronic stage, macrophages shift toward an anti-inflammatory state (25). This switch suggests a complex interplay within the immune system. However, there is currently no *in vivo* study exploring the potential inhibitory role of macrophages in HIV pathogenesis.

Humanized (hu) mice are based on the transplantation of human hematopoietic progenitor and stem cells (HSPCs) into immunocompromised mice, which results in the reconstitution of human immune cells (26). When infected with HIV, these mice can maintain disseminated infection and represent an invaluable tool for HIV research (27). In this study, we investigated whether macrophages have an anti- or pro-viral effect on HIV replication in HIV-infected hu NOD *scid* Gamma (NSG) mice by creating a new hu mouse model, in which we were able to selectively deplete human myeloid cells without perturbing any other immune subset. We found that myeloid cell depletion resulted in a significant increase in HV replication in these HIV-infected mice. Using co-culture assays, cytokine analysis and transcriptomic analysis, we showed that macrophages upregulate IFN-stimulated genes (ISGs) and production of HIV co-receptor antagonists which may be the reason for the restriction of HIV replication. These data suggest a novel role of macrophages in HIV infection, which has long been overlooked.

## 2 Materials and methods

### 2.1 Generation of humanized mice

Hu mice were generated as previously described by Audige et al. (28). In brief, immunodeficient NOD.Cg-*Prkdc^scid^ Il2rg^tm1Wjl^*/SzJ (NSG) mice were obtained from Charles River Laboratories, and bred and maintained at the Laboratory Animal Service Center of the University of Zurich. Newborn mice were irradiated with 1Gy 1-3 days after birth and subsequently transplanted with 1.5 ± 0.5 x10^5^ CD34+ cells via intrahepatic injection. We checked the degree of human cell engraftment at approximately 16 weeks of age by staining peripheral blood for the pan-human marker CD45.

### 2.2 Cells and reagents

HEK 293T cells (ATCC, Cat#: CRL-1573) and TZM-bl cells (NIH AIDS Reagents Program, GenBank: ARP-5011) were cultured in Dulbecco’s Modified Eagle Medium (DMEM) (Sigma-Aldrich, Cat#: D5796) supplemented with 10% fetal bovine serum (FBS) (Gibco, Cat#: 26140), 1% 2mM L-glutamine and 1% (Thermo Fischer Scientific, Cat#: 25030081), Penicillin/Streptomycin (Thermo Fischer Scientific 15140148)). PBMCs were isolated from buffy coats via gradient centrifugation, using Greiner Bio-One LeucoSEP™ Polypropylene Tubes (Thermo Fisher scientific, Cat#: Z642843) filled with 15 ml Lymphoprep™ (Stemcell technologies, Cat#: 07861). For both CD8+ T cell and NK cell depletion (**Supplementary Figure S14**), we performed a negative selection with magnetic bead sorting, either CD8 MicroBeads, human (Miltenyi biotec, Cat#: 130-045-201) or NK cell isolation kit, human (Miltenyi biotec, Cat#: 130-092-657) according to manufacturer protocol (29). Non targeted fraction was used for experimentation. PBMCs were cultured in RPMI-1640 medium supplemented with 10% FBS, interleukin-2 (rhIL-2) (Immunotools, Cat#: 11340027) at 10 U/ml, 2mM L-glutamine, and 1% Penicillin–Streptomycin. All cells were incubated at 37°C and 5% CO_2_.

### 2.3 Virus production and titration

Both the CCR5-tropic HIV YU-2 and the CXCR4-tropic HIV NL4-3 molecular clones from NIH AIDS Reagent Program were transfected into HEK 293T cells using polyethylenimine (PEI, Thermo Fisher Scientific, Cat# BMS1003-A). Culture supernatants were harvested 48 hrs later, filtrated (0.22µM, Steriflip, EMD Millipore, Cat#: SE1M179M6) and then stored at -80°C until further use. Tissue culture infectious dose 50 (TCID_50_) of the viral stocks was determined by infecting TZM-bl cells. 48 hrs after infection, cells were lysed with Passive Lysis Buffer (Cat#: E1941, Promega) and used for measuring the emitted luminescence (Cat#: E1501, Promega) with GloMax Navigator Microplate Luminometer (Promega, Cat#: GM2000). The cut-off for counting a well as HIV+ was 2.5x the background relative light units (RLU). Virus controls had to reach ≥10x the background RLU. TCID_50_ was calculated according to the Reed Muench method (30).

### 2.4 *In vivo* HIV infection

HuNSG mice with human engraftment level of CD45+ cells > 5% were infected with HIV-1 YU-2 at a TCID_50_ of 2x10^5^ via intraperitoneal (i.p.) injection approximately one week after checking human engraftment level. Viral dissemination and replication were investigated in plasma of infected mice starting from 4 weeks after infection.

### 2.5 Co-culture of MDMs with *ex vivo* HIV-infected PBMCs

In 6-well plates, PBMCs from 3 different donors, were plated at a density of 1x10^6^ per well, in triplicate. PBMCs were activated for 3 days in RPMI-1640 supplemented with 2 ng/ml phytohemagglutinin (PHA-P) (ThermoFisher, Cat#: 543613) and 50 ng/ml rhIL-2, 10% FBS, 1% penicillin-streptomycin. After 3 days, the medium was changed to RPMI-1640 supplemented with 10 ng/ml rhIL-2, 10% FBS, 1% penicillin-streptomycin. Activated PBMCs were infected with HIV-1 NL4-3 at a multiplicity of infection (MOI) of 0.01 for 12 hrs and cultured for an additional 2 days before co-cultivation with autologous MDMs. For obtaining the autologous MDMs, monocytes were isolated from PBMCs via magnetic-beads sorting (CD14 MicroBeads, human) (Miltenyi Biotec, Cat#: 130-050-201), according to manufacturer protocol (31). CD14 monocytes were differentiated for 6 days in RPMI-1640 medium supplemented with 10% human AB serum (Sigma Aldrich, Cat#: H4522) at a density of 1x10^6^ per well in a F-bottom 6-well plate (Starstedt, Cat#: 83.3920). Pro-inflammatory M1 polarization was achieved by adding 20 ng/ml rhIFNγ (Immunotools, Cat#: 11343534) and 100 ng/ml LPS (Sigma Aldrich, Cat#: L2387) for an additional 2 days, and anti-inflammatory M2 polarization by adding 20 ng/ml rhIL-4 (Immunotools, Cat#: 11340043). In parallel, we kept unstimulated MDMs (M0) in order to have resting macrophages. *Ex vivo* HIV-1 infected PBMCs were eventually co-cultured with either pro- or anti-inflammatory, or resting macrophages, derived from the same donor. Co-cultures were performed in two separate ways, either in direct contact (cell-to-cell) or separated by a trans well with 1μm pores (Starstedt TC inserts, Cat#: 83.3920).

### 2.6 TZM-bl Luciferase assay

TZM-bl cells were seeded at a density of 1x10^4^ cells per well in a 96-well F-bottom microplate (Greiner Bio-One, Cat#: 655090). 50 μl of co-culture supernatant was added in triplicate, per well, per sample. After 48 hrs incubation, Luciferase Assay System (Promega, Cat#: E1501) was used according to manufacturer protocol (32), for mammalian cells. Results were acquired using the GloMax Navigator Microplate Luminometer (Promega, Cat#: GM2000), setting exposure to 1 sec.

### 2.7 *In vivo* macrophage depletion using clodronate liposomes

Depletion of murine and human phagocytes was performed via intraperitoneal (i.p.) injections of clodronate liposomes. The loading dose was 0.03 mg/g body weight, followed by weekly injections with a maintenance dose of 0.015 mg/g body weight.

### 2.8 Lentiviral production

Third generation lentiviral particles (33) were produced in HEK 293T cells. Briefly, transfection mix was prepared in Opti-MEM medium (ThermoFisher Scientific, Cat#: 11058021) by adding 14.5 µg of pDEST_SP107-iCasp9GFP_pGK-NGFR (**Supplementary Figure S7A**) lentiviral plasmid, 8.8 µg pSYNGP plasmid, 4 µg RSV-rev plasmid and 4.8 µg pVSV plasmid per ml of transfection mix. The polycation, PEI was added, to the mix in a 1:6 dilution. The transfection mix was incubated 20 min at room temperature (RT) and added to the cells. Medium was changed after 6 hrs with Opti-MEM supplemented with 5mM sodium butyrate, harvested 48 hrs after transfection and spun down at 300g for 5 min. to remove cell debris, and sterile filtered. Lentiviral particles were concentrated via Amicon Centrifugal Filter Unit (Sigma Aldrich, Cat#: UFC901096), according to manufacturer protocols (34). Lentiviral production was validated via Lenti-X GoStix Plus (Takara, Cat#: 631280), according to manufacturer protocol (35), and stored at -80°C. Lentiviral titration was performed by transduction of 6 serial dilutions in HEK 293T cells.

### 2.9 PBMCs transduction

PBMCs were transduced with lentiviral particles by seeding at a MOI of 5 for 12 hrs at a density of 2x10^5^ cells per well in a 24-well plate. To promote transduction, we also added polybrene (Sigma Aldrich, Cat#: TR-1003) at a 1:1000 dilution. Transduced PBMCs were cultured 36 hrs prior to transduction evaluation via flow cytometry and treatment with Rimiducid (AP1903) (MedChemExpress, Cat#: 195514-63-7).

### 2.10 HSPCs isolation and generation of recombinant HSPCs

We isolated white blood cells from the umbilical cord blood using Greiner Bio-One LeucoSEP™ Polypropylene Tubes filled with 15 ml Lymphoprep™ and then CD34+ cells via magnetic beads sorting (CD34 MicroBeads, human) (Miltenyi biotec, Cat#: 130-046-702) according to manufacturer protocol (29). CD34+ cells were either stored in liquid nitrogen in 90% FBS and 10% DMSO or were immediately transduced. For the transduction, CD34+ cells were cultured in Isove’s modified eagle’s medium (Thermo Fisher Scientific, Cat#: 12440053) supplemented with 20% BIT 9500 serum substitute (Stemcell technologies, Cat#: 09500), 10 μg/ml human low-density lipoprotein (Stemcell technologies, Cat#: 02698), 0.1mM 2-mercaptoethanol (Sigma Aldrich, Cat#: 63689-25ML-F), 0.1mM Glutamax 500 (Glibco, Cat#: 35050087), 100 ng/ml FLT3-L, 100 ng/ml SCF, 20 ng/ml IL-3, 20 ng/ml IL-6, 20 ng/ml G-CSF (all Immunotools, Cat#: 11343303, 11343325, 11340033, 11340064, 11343133) and UM171 35nM. CD34+ cells were transduced 12 hrs after isolation at a MOI of 5, in the presence of LentiBlast Premium (1:500) (OZ Biosciences, Cat#: LBPX500) and medium was changed 16 hrs later. Transduced CD34+ cells were cultured for 5 days. NGFR+, CD34+ cells were sorted via magnetic beads sorting (CD271 (NGFR) MicroBeads, human) (Miltenyi biotec, Cat#: 130-099-023). Sorted cells were kept at -80°C in 90% FBS and 10% dimethyl sulfoxide (DMSO (Sigma Aldrich, Cat#: 67-68-5) until further use.

### 2.11 AP1903 reconstitution and treatments

AP1903 was reconstituted in DMSO with a final concentration of 10mM for cell culture experiments, as per manufacturer protocol (36). Cells were treated with 10nM. AP1903 for *in vivo* treatment, reconstituted in 10% DMSO and 90% corn oil to achieve a concentration of 2.5 mg/ml, as per manufacturer protocol (36). iHMD-NSG mice were treated intravenously (i.v.) via tail vain injection at a concentration of 5 mg/kg. All dilutions of the stock solution were made in phosphate buffered saline (PBS) (Gibco, Cat#: 10010023).

### 2.12 Viral load assay

60µl of plasma was collected from the blood by low-speed centrifugation and used for RNA isolation (QIAmp viral isolation kit, Qiagen, Cat#: 52906). Isolated RNA was eluted in 30µl AVE buffer of which 12µl was used for reverse transcription (RT) (iScript Select cDNA synthesis kit, BioRad, Cat#: 1708897). The reverse primer used for cDNA synthesis was skcc (TACTAGTAGTTCCTGCTATGTCACTTCC), which binds to the gag region. qPCR was carried out by using DreamTaq™ Hot Start PCR Master Mix (Thermo Fisher Scientific, Cat#: K9012), skcc (reverse) and ts5’gag (CAAGCAGCCATGCAAATGTTAAAAGA) (forward) primers, and FAM-BHQ1 probe mf319 (TGCAGCTTCCTCATTGATGGT). 10µl of viral cDNA was used in the qPCR reaction. Standards (7.13x10^7^ – 200 copies per ml) were generated by using an HIV-1 culture supernatant with a known copy number (determined using the COBAS TaqMan HIV-1 Test (Roche)).

### 2.13 Cytokine evaluation

Serum concentration of cytokines and soluble cytokine receptors were evaluated in serum samples from iHMD-NSG and cell culture supernatant. The following cytokines were evaluated: IL-1α, IL-1RA, IL-1β, IL-2, IL-4, IL-5, IL-6, IL-7, IL-9, IL-10, IL-12p70, IL-13, IL-15, IL-17A, IL-18, IL-21, IL -22, IL-23, IL-27, IL-31, IFN-α, IFN-γ and TNF, chemokines, i.e., CCL2, CCL3, CCL4, CCL5, CCL11, CXCL1, CXCL8, CXCL9, CXCL10, CXCL12, CXCL13 and TNF-β and growth factors, i.e., NGF-β, BDNF, EGF, FGF-2, HGF, LIF, PDGF-BB, PlGF-1, SCF, VEGF-A, VEGF-D, BAFF, GM-CSF and G-CSF. Cytokine concentrations were determined by multiplex bead assay as previously described by Amelio at al. (37). The upper normal values for each marker were defined based on the results obtained in the 450 sera collected from healthy individuals (mean + 2 standard deviations).

### 2.14 Flow cytometry

All Cat# for all antibodies listed in **Supplementary Table S1**.

#### 2.14.1 Staining of *ex vivo* samples

HSPCs were stained for human cell surface markers CD34, CD3, NGFR. PBMCs were stained for human cell surface markers CD3, CD4, CD8, CD14, CD19, NGFR (all BioLegend). Immune phenotyping of MDMs was performed on polarized macrophages by staining for human surface markers HLA-DR, CD38, CD86, CD80, CD11c, CD209, D206, CD163, CD33, CD3, CD19, CD56, CD66b (all BioLegend). Macrophages were detached from the culture plate by pipetting with ice-cold PBS supplemented with 2mM EDTA (Gibco, Cat#: 17892) for approximately 10 min per well. Compensation controls were performed using Ultra Comp eBeads (Thermo Fisher Scientific, Cat#: 01-2222-42). Washing and reagent dilutions were done in PBS or FACS buffer (PBS containing 2% FBS and 0.05% sodium azide). Before acquisition, cells were fixed with 1% paraformaldehyde (PFA) (Sigma, Cat#: P6148)

#### 2.14.2 Staining of *in vivo* samples

Immunophenotypic analysis of peripheral blood, splenocytes, hepatocytes, bone marrow leukocytes, lung leucocytes and peritoneal cells was performed to monitor and determine human engraftment, immune activation and the phenotype of multiple cell lineages. Peripheral blood was collected into EDTA tubes. 20μl whole blood were used to determine white blood cell counts. For immunophenotypic analysis, 80-100μl of whole blood were used. Erythrocyte removal from stained whole blood was performed with BD FACS Lysing Solution (BD Biosciences, Cat#: 349202). At necropsy, 500μl of blood were withdrawn. In this instance, leukocytes were extracted by gradient centrifugation in Lymphoprep™. Bone marrow cells were obtained from mouse femurs by flushing the bone marrow out with a 300μl syringe. Single cell suspensions of splenocytes, hepatocytes, lung leukocytes and lymph node leukocytes were obtained by pressing the organs through 70µm nylon mesh. Gradient centrifugation of splenocytes, hepatocytes and lung lymphocytes, was performed with Lymphoprep™, for separation from erythrocytes. In order to extract tissue resident macrophages, lung, liver and spleen tissue samples were reduced and incubated in Collagenase solution (HBSS) (Thermo Fisher, Cat#: 88284), 10% FBS, Collagenase type 4 (0.4 mg/ml) (Sigma Aldrich, Cat#: C4-22-1G)), for 45 min at 37°CC, prior to homogenization through a 70μl cell strainer. 20μl of cell suspension was used to determine the total tissue cell count. 1x10^6^ cells per tissue were stained for flow cytometry. Different staining protocols were used in different experiments. Depending on the staining, the following surface markers were used: murine CD45, F4/80, CD11b, Ly6C, Ly6G; human CD45, CD3, CD4, CD8, CD19, CD33, NKp46, CD56, CD14, CD16, CD19, CD86, CD11b, NGFR, CD66b, HLA-DR, CD38, CD86, CD80, CD11c, CD209, CD206, CD163, CD33 (all BioLegend, **Supplementary Table S1**). Furthermore, splenocytes and bone marrow cells were stained for human cell surface markers CD45, CD3, CD4, CD8, CD11b (all BioLegend) and for HIV-1 core antigen intracellular marker p24 (Beckman Coulter). Intracellular staining was performed with fixation/permeabilization kit (BD Biosciences), according to manufacturer protocol (38). Compensations for intracellular p24 signal were performed in ACH-2 cells (NIH AIDS program) activated via TNFα stimulation (0.5 ng/ml). The Zombie NIR fixable Viability Kit (BioLegend, Cat#: 423107) was included in all multicolor staining panels, and compensation controls were performed using Ultra Comp eBeads. Washing and reagent dilutions were done in PBS or FACS buffer. Before acquisition, cells were fixed with 1% paraformaldehyde. All samples were acquired at BD LSR II Fortessa (BD Bioscience). Data were analyzed using FlowJo software 10.8.1 (TreeStar).

### 2.15 Bulk RNA sequencing

RNA sequencing was performed on either CD4+ T cells (isolated from PBMCs) or M1-polarized MDMs, from either *ex vivo* co-culture or monocultures. In fact, we followed exactly the same experimental design as described in the paragraph “Co-culture of MDMs with *ex vivo* HIV-infected PBMCs”. As the most potent HIV inhibition was observed at 72 hours after co-culturing, we harvested the two cell types at this time point. We performed this experiment with three donors, with three replicates from each donor. For CD4+ T cells, both HIV infected and uninfected cells were isolated after 72 hours of either monoculture or co-culture with MDMs via magnetic bead sorting (CD4 MicroBeads, human, Miltenyi biotec, Cat #: 130-097-048), according to manufacturer protocol (29). We performed two cycles of magnetic beads sorting in parallel, aiming to maximize population purity. Plates with adherent MDMs were washed 3 times with PBS to remove PBMCs residues. RNA from both CD4+ T cells and MDMs was isolated with RNeasy mini kit (Qiagen, Cat#: 74106), according to manufacturer protocol (39). All samples were sequenced paired-end 150bp on the NovaSeqX. The library prep method used is “Illumina Stranded mRNA Prep, Ligation” as per manufacturer protocol.

#### 2.15.1 Cluster generation and sequencing

The Novaseq X Plus (Illumina, Inc, California, USA) was used for cluster generation and sequencing according to standard protocol. Sequencing were paired end at 2 X150 bp or single end 100 bp.

#### 2.15.2 Library preparation

The quality of the isolated RNA was determined with a Fragment Analyzer (Agilent, Santa Clara, California, USA). Only those samples with a 260 nm/280 nm ratio between 1.8–2.1 and a 28S/18S ratio within 1.5–2 were further processed. Illumina Stranded mRNA Prep, Ligation (Illumina, Inc, California, USA) was used in the succeeding steps. Briefly, total RNA samples (100-1000 ng) were poly A enriched and then reverse-transcribed into double-stranded cDNA. The cDNA samples were fragmented, end-repaired and adenylated before ligation of TruSeq adapters containing unique dual indices (UDI) for multiplexing. Fragments containing TruSeq adapters on both ends were selectively enriched with PCR. The quality and quantity of the enriched libraries were validated using the Fragment Analyzer (Agilent, Santa Clara, California, USA). The product is a smear with an average fragment size of approximately 260 bp. The libraries were normalized to 10nM in Tris-Cl 10 mM, pH8.5 with 0.1% Tween 20.

#### 2.15.3 The RNA-seq data analysis

The raw reads were first cleaned by removing adapter sequences and poly-x sequences (> 9 nt used for detection) using fastp (Version 0.23.4) (40). Reads with length <18nt after trimming were additionally filtered out. Sequence pseudo alignment of the resulting high-quality reads to the Human reference genome (build GRCh38.p13) and quantification of gene level expression (gene model definition from GENCODE release 42) was carried out using Kallisto (Version 0.46.1) (41). To detect differentially expressed genes we used the glm approach implemented in the software package DESeq2 (R version: 4.3.2, DESeq2 version: 1.42.0) (42). For comparisons with additional baselines, the glm approach implemented in EdgeR was used (R version: 4.3.2, EdgeR version: 4.0.1) (43). Terms with a false-discovery rate (FDR) < 0.05 were considered significant. Genes showing altered expression with FDR-adjusted p-value <= 0.01 and log2 ratio >= 0.5 were considered significant. Over-representation analysis (ORA) was conducted based on differentially expressed genes with FDR-adjusted p-value < 0.01 using clusterProfiler (Version 4.10.0) (44). Raw data can be access in GEO public repository: https://www.ncbi.nlm.nih.gov/geo/query/acc.cgi?&acc=GSE269285.

### 2.16 Statistical analysis

All statistical analyses beside bulk RNA sequencing were performed with GraphPad Prism 8.0 software (GraphPad Software, Inc., La Jolla, CA). The individual statistical test is indicated in each respective figure legend.

### 2.17 Study approvals

All animal experiments were approved by the Cantonal Veterinary Office (license ZH181/17; Amendment 28395 of ZH243/16; ZH081/21). Human cord blood collection was approved by the Ethical Committee of the Kanton Zurich (#EK1103) and collected with the informed consent from the donors. Buffy coats were anonymously provided by the Blood Donation Service Zurich, Swiss Red Cross, Schlieren.

## 3 Results

### 3.1 HIV infection leads to myeloid cell activation and expansion of pro-inflammatory subset in huNSG mice

**Figure 1:**
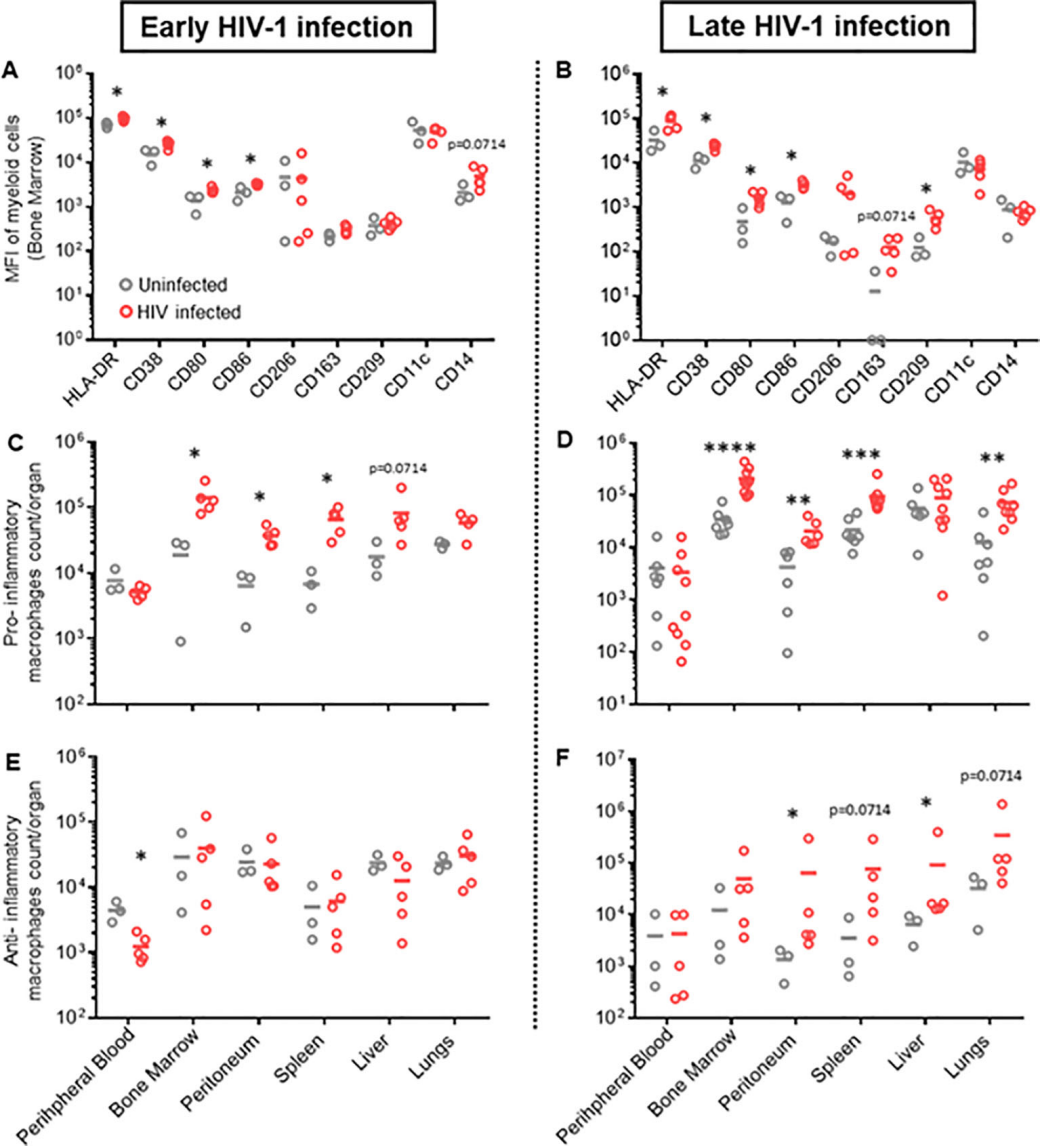
Different phenotypic patterns of human myeloid cells in early and late HIV infection in hu NSG mice. Humanized NSG mice were either infected with HIV-1 YU-2 at TCID50 2*105 (HIV+) or left uninfected (HIV-). **(A, C, E)**. Half of the mice from each group were euthanized either 4 weeks (early HIV-1 infection, left panel) or **(B, D, F)** 12 weeks after infection (late HIV infection, right panel). Leukocytes were isolated from bone marrow, peritoneum, spleen, liver, lungs and peripheral blood. Cells were characterized via flow cytometry. **(A, B)** MFI of multiple myeloid surface markers, calculated for myeloid cells of bone marrow for early and late HIV infection (Mean; n HIV-=3; n HIV+=5) (see also **Supplementary Figure S3**). **(C, D)** Total count/organ of human CD45+ cells with phenotype CD33+, CD3-, CD19-, CD56-, HLA-DR+, CD38+, CD80+, CD86+, were calculated by using cell population frequencies, obtained by flow cytometry, on total cells processed per sample (Mean) **(C)** n HIV-=3; n HIV+=5; **(D)** n HIV-=6; n HIV+=9). **(E, F)** Total count/organ of human CD45+ cells with phenotype CD33+, CD3-, CD19-, CD56-, CD206+, CD163+, were calculated by using cell population frequencies, obtained by flow cytometry, on total cells processed per sample (Mean; n HIV-=3; n HIV+=5). Statistics by Mann-Whitney **(A–F)**. p-value: *<0.05; **<0.01; ***<0.001; ****<0.0001.

Natural HIV infection in humans results in an activated and exhausted phenotype in immune cells, which has been extensively shown for T-cells (45). However, *in vivo* data on myeloid cell activation during HIV infection are scarce. Therefore, we performed a phenotypical characterization of human mononuclear phagocytes in multiple organs of HIV-infected and uninfected huNSG mice at four (early HIV-1 infection) and twelve weeks (late HIV-1 infection) post-infection. We found an upregulation of pro-inflammatory markers, *i.e.*, HLA-DR, CD38, CD86, and CD80, during early HIV-1 infection stage in several organs (**Figure 1A**, **Supplementary Figure S1**), which persisted into the late infection stages (**Figure 1B**, **Supplementary Figure S2**). In contrast, we found no differences in anti-inflammatory markers, CD206, CD163 and CD209, in early HIV infection between the two groups, while they increased in the later infection stage (**Figures 1A, B**, **Supplementary Figures S2**, **S3**). Furthermore, we observed a higher number of specific subsets of human mononuclear phagocytes defined by their expression of pro-inflammatory markers, HLA-DR, CD38, CD80 and CD86 (**Supplementary Figure S3**), in HIV-infected mice during acute and chronic infection in bone marrow, peritoneum, lungs and spleen (**Figures 1C, D**). An increase in human mononuclear phagocytes expressing anti-inflammatory markers, CD163, CD206 and CD209, was observed in all organs, but only during late HIV infection (**Figures 1E, F**). In addition, we observed a higher number of monocytes, only in specific tissues, during both early and late HIV infection (**Supplementary Figure S4A**), while no difference was observed for dendritic cells (**Supplementary Figure S4B**). Taken together, these results showed that HIV infection induces strong activation of human mononuclear phagocytes in humanized NSG mice and underscored their plasticity and functionality in huNSG mice. We also noted differences in immune activation at early vs late time points, regardless of HIV infection. This difference was actually expected since the human lymphatic system of humanized mice is known to undergo dynamic changes overtime (46).

### 3.2 Clodronate-mediated depletion of macrophages results in substantially increased HIV replication in HIV-infected hu mice

**Figure 2:**
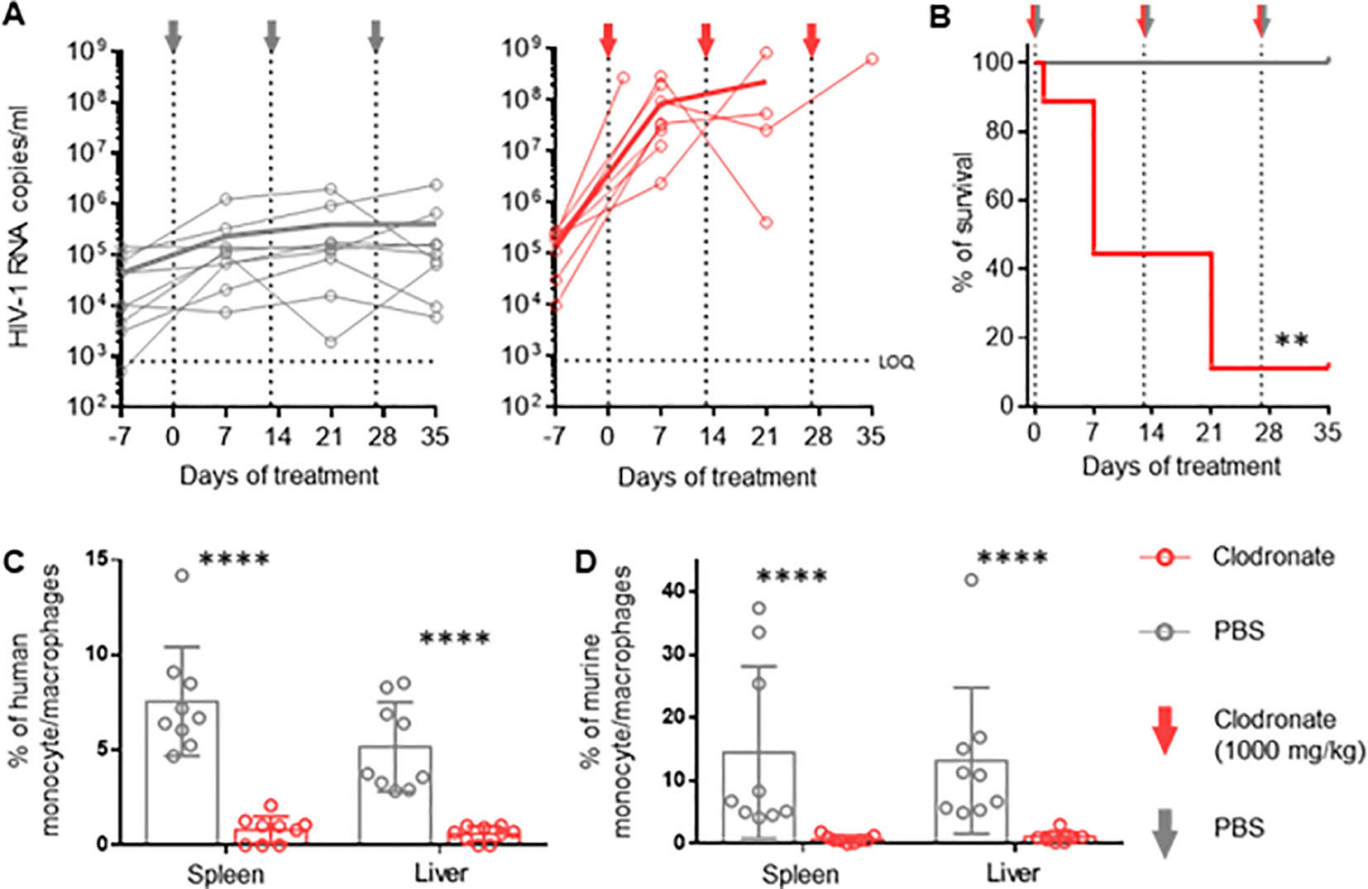
Vigorous increase in HIV replication in hu mice following clodronate mediated depletion of macrophages RNA was extracted from plasma obtained from HIV-1-infected humanized NSG mice treated with clodronate-liposomes (n=9) or PBS (n=9) at day 7, 21 and 35. **(A)** Each circle represents an individual mouse; HIV-1 RNA copies were measured via in-house RT-qPCR. Thick lines represent the mean of each respective group. **(B)** Treatment toxicity was established via Kaplan-Meier survival analysis. **(C, D)** Flow cytometric analysis of leukocytes from spleen and liver of clodronate and mock treated animals. Both human (hCD45+, hCD3-, hCD19-, hCD33+, hCD68+, hCD163) **(C)** and murine (mCD45+, Ly6G-, Ly6C-, CD11b+, F4/80+) **(D)** myeloid cells were analyzed (Mean and standard deviation). Mantel-Cox test **(B)**; Mann-Whitney **(C, D)**. p-value: **<0.01; ****<0.0001.

In order to evaluate the role of activated mononuclear phagocytes in HIV infection in huNSG mice, we depleted these cells in HIV-infected mice using clodronate-liposomes (47). One week after treatment, HIV viral load increased up to three logs in the clodronate-treated animals and remained higher than in the control group throughout the experiment (**Figure 2A**), suggesting that phagocytes are involved in the control of HIV-1 replication over time. Notably, hu mice harbor murine and human macrophages (48) (**Supplementary Figure S5A**). However, clodronate-mediated depletion is not species-specific (**Figures 2C, D**) making it impossible to conclude the role of human phagocytes in the system.

Interestingly, we observed higher CD4+ T cell counts (contrary to CD8+ T cells and NK cells) in the spleen and liver of clodronate-treated animals compared to control animals (**Supplementary Figures S6C–E**). It is conceivable that the depletion of murine macrophages liberated space for human T-cell expansion, which in turn provided a larger HIV target cell population, at least partially explaining the increase in HIV viral load. Furthermore, clodronate treatment showed high toxicity very early on, resulting in a very low survival rate during the first two weeks (**Figure 2B**). The extent to which toxicity leads to immune activation and, thus, higher HIV replication remains unanswered as well. The specific role of human macrophages herein remains to be elucidated.

### 3.3 A novel humanized mouse model enabling targeted *in vivo* depletion of human myeloid cells

To investigate the role of mononuclear phagocytes in HIV infection *in vivo*, we created a novel hu mouse model that allows for selective depletion of human myeloid cells. The basic concept was to transplant NSG mice with HSPCs carrying the gene of an apoptosis inducible caspase 9 (iCasp9), which is only active in myeloid cells. Therefore, we generated a lentiviral-based construct that encodes the iCasp9 (49) under the control of a myeloid-specific synthetic promoter, p47-SP107 (50) (**Supplementary Figure S7A**). iCasp9 apoptosis activity is dependent upon its homodimerization, which is triggered by the otherwise inert molecule AP1903 (49). To identify cells expressing iCasp9, we cloned a GFP downstream of iCasp9, separated by a cleavage peptide. Furthermore, we inserted the truncated neural growth factor receptor (NFGR) under the control of the constitutive promoter pGK to identify and sort the successfully transduced HSCs, which were used for transplanting newborn mice.

**Figure 3:**
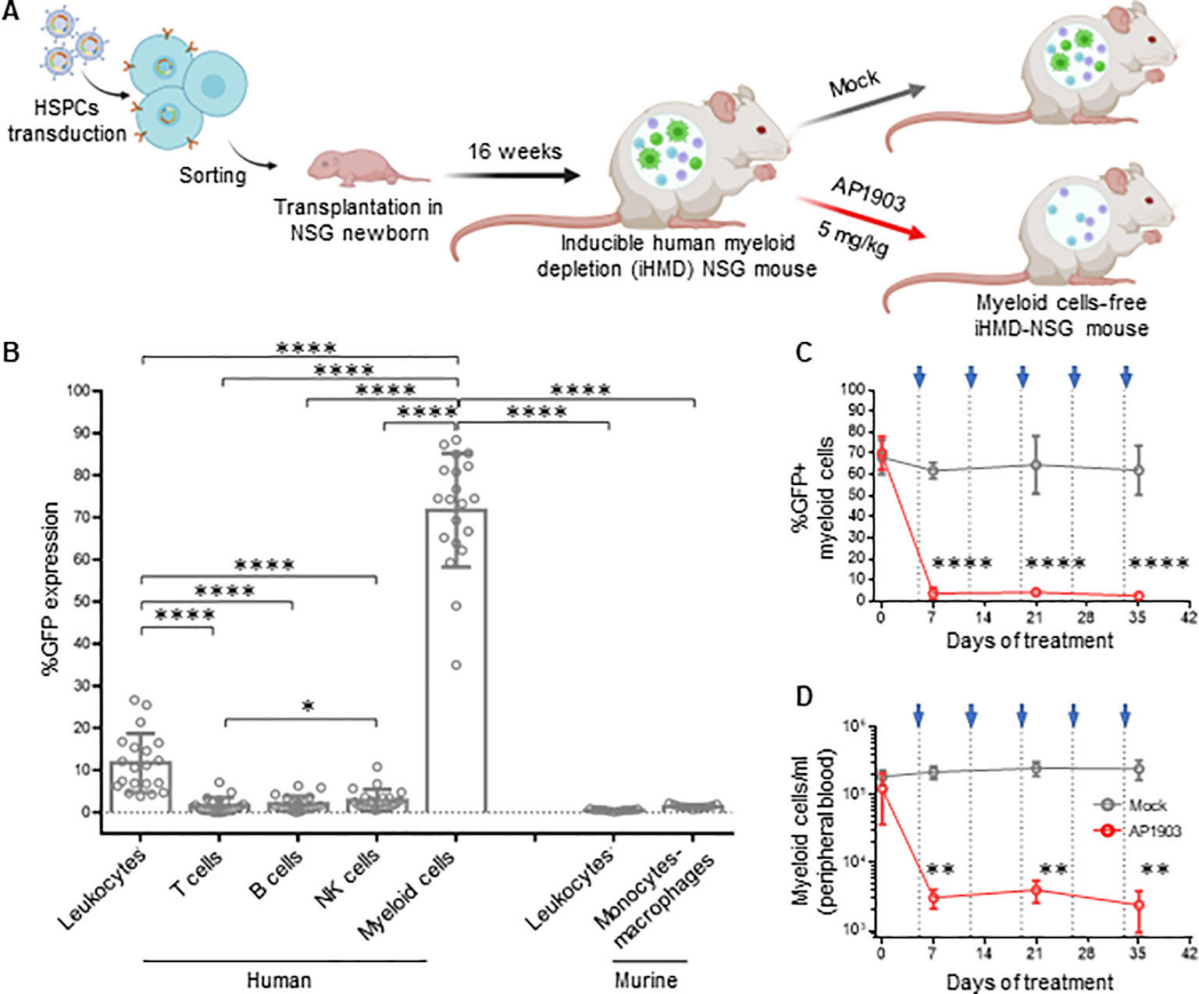
Efficient depletion of myeloid cells in iHMD-NSG mice. **(A)** Schematics for the generation and functionality testing of iHMD-NSG mice. BioRender.com/g70c130 Engraftment in iHMD NSG mice was evaluated 16 weeks post transplantation. Engrafted mice were treated once a week for 5 weeks. **(B)** GFP expression in human leukocytes from 3 independent cohorts (n=20) and murine leukocytes from 2 (n=11) (Mean and standard deviation). **(C)** Flow cytometry analysis of GFP expression in human myeloid cells from peripheral blood of iHMD-NSG mice. AP1903 5mg/kg (red, n=4) and mock (grey, n=3) (Mean and standard deviation). **(D)** Myeloid cell count overtime for both treatments, was calculated by using cell population frequencies, obtained by flow cytometry, on total cells processed per peripheral blood sample (Mean and standard deviation). Statistics by Mann-Whitney **(B)**. Two-way ANOVA **(C, D)**. p-value: *<0.05; **<0.01; ***<0.001; ****<0.0001.

We first tested the construct *ex vivo* in peripheral blood mononuclear cells (PBMCs). Upon transduction, we observed NGFR expression in all cell types, while GFP expression was mainly observed in monocytes (**Supplementary Figures S7B, C**). This result confirmed the myeloid specificity of the p47-SP107 promoter. Upon treatment with AP1903, >90% of GFP+ cells had undergone apoptosis, demonstrating the high efficiency of iCasp9. We speculate that the higher NGFR expression in monocytes, compared to other cell types, is a result of the high p47-SP107 activity in myeloid cells (50). NGFR expression in B and T cells, as well as their absolute count remained unchanged irrespective of AP1903 (**Supplementary Figure S7D**). Next, HSPCs were transduced with the lentiviral construct and sorted for the NGFR+ HSPCs (**Supplementary Figure S8A**). We obtained a population of >95% of genetically modified cells, which were used for the humanization of NSG mice (**Figure 3A**). Human engraftment level in these mice transplanted was lower when compared to mice transplanted with wild-type HSCPs (**Supplementary Figure S8B**). However, the human leukocyte distribution was independent of using genetically modified or wild-type HSPCs (**Supplementary Figure S8C**). As we expected, we observed high GFP expression in human myeloid cells (71.7% ± 13.49%), very low expression in NK cells (2.90% ± 2.53%), and negligible expression in T cells, B cells and murine cells obtained from peripheral blood samples (**Figure 3B**). These results confirmed the specificity of our lentiviral construct. The GFP expression corresponded to the average 10% of myeloid cells typically present in the total leukocyte population (51).

A single injection of AP1903 triggered apoptosis in >90% of the GFP+ cells within 24h in bone marrow, spleen, liver and peripheral blood (**Supplementary Figure S9A**). In addition, weekly injections of AP1903 were well tolerated (**Supplementary Figure S9B**) and resulted in consistently low GFP expression and absolute count of myeloid cells (**Figures 3C, D**). GFP expression and cell counts in the different organs were comparable to peripheral white blood cells (**Supplementary Figures S9C, D**). In addition, the longitudinal analysis of human leukocytes showed that AP1903 had no impact on the human graft (**Supplementary Figure S9E**). Therefore, with this inducible human myeloid depletion (iHMD) model, we were able to selectively deplete human myeloid cells without targeting other human cell types.

### 3.4 Significant increase in HIV replication as a result of human myeloid cell depletion

**Figure 4:**
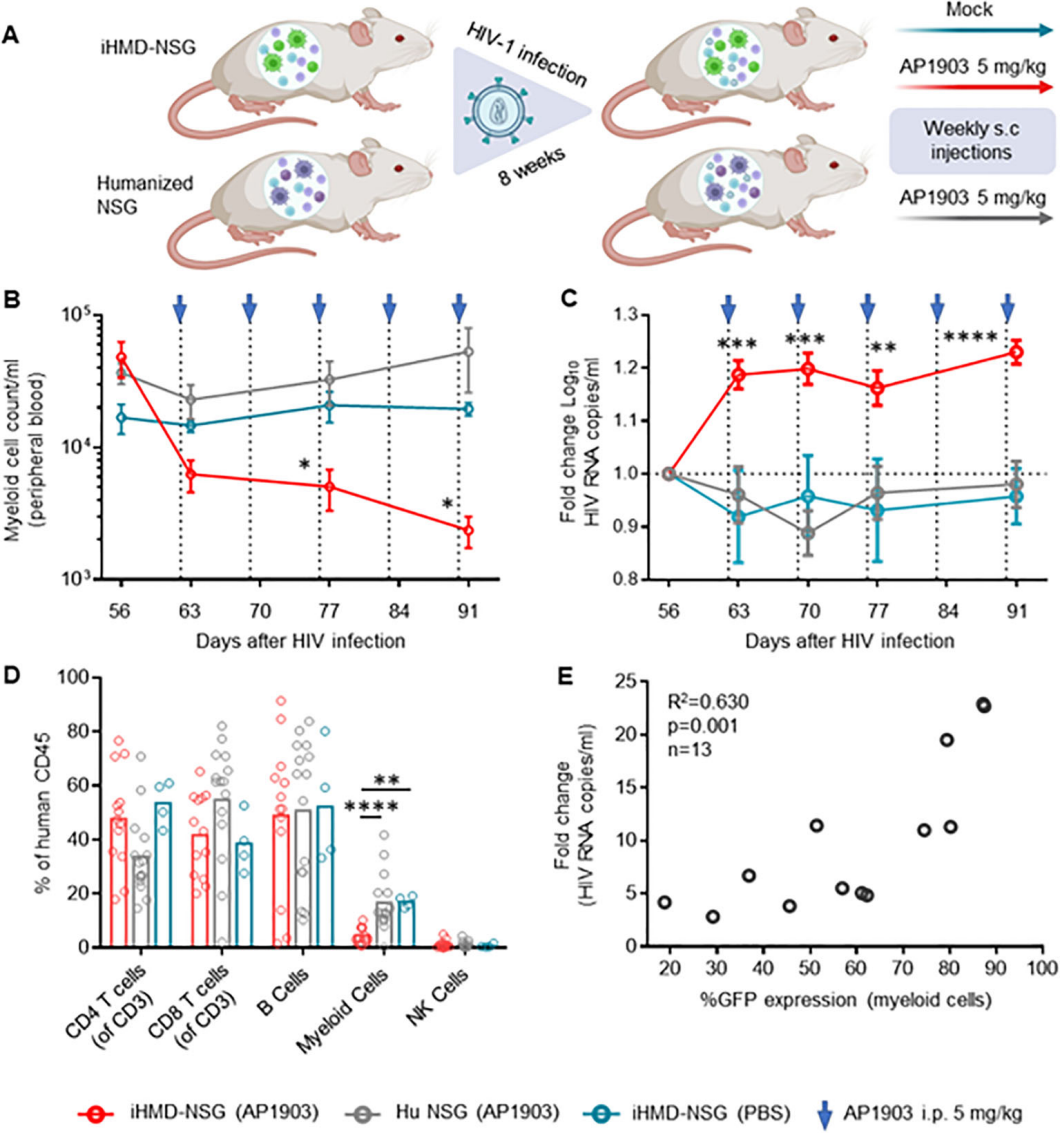
Depletion of myeloid cells results in a vigorous increase of HIV viral load. **(A)** Schematics of experimental procedure, BioRender.com/m34c552. Engrafted mice were infected with HIV YU-2 for 8 weeks. HIV infected mice were treated once a week for 5 weeks **(B)** Myeloid cell count overtime for the three treatment regimens overtime, were calculated by using cell population frequencies, obtained by flow cytometry, on total cells processed per sample (Mean and standard error of the mean). **(C)** Fold change in viral load compared to pre-treatment baseline VL at day 56, via RT-qPCR. Results from 3 independent studies are shown: Hu NSG mice treated with AP1903 (5mg/kg) (n=15), iHMD-NSG treated with either AP1903 (5mg/kg) (n=13) or PBS (n=4) (Mean and standard error of the mean). **(D)** Leukocytes frequencies distribution in the three treatment regimens, measured vial flow cytometry at euthanasia (Mean). **(E)** Linear regression analysis between average fold change in HIV RNA copies/ml of peripheral blood and GFP expression in myeloid cells prior AP1903 treatment in iHMD-NSG mice. Statistic by two-way ANOVA **(B, C)**, Mann-Whitney **(D)**. p-value: *<0.05; **<0.01; ***<0.001; ****<0.0001. Linear regression analysis **(E)**.

We generated iHMD-NSG or huNSG mice with HSPCs from three different donors to consider donor-specific differences. Both iHMD-NSG and huNSG mice were infected with HIV for eight weeks to achieve stable viremia over time. iHMD-NSG mice were then treated with weekly injections of either AP1903 (5mg/kg) or PBS, and huNSG mice with AP1903 (**Figure 4A**). In fact, we observed a significant increase in HIV RNA copy numbers in iHMD-NSG mice after the first dose of AP1903, which persisted throughout the treatment period (**Figure 4C**, **Supplementary Figure S10A**). We confirmed that AP1903 in iHMD-NSG mice resulted in vigorous depletion of myeloid cells in peripheral blood, bone marrow, liver and spleen (**Figures 4B, D**, **Supplementary Figure S10D**). No effect was observed in either PBS-treated iHMD-NSG mice or AP1903-treated huNSG mice. Furthermore, we observed a correlation between the initial %GFP expression in myeloid cells and the increase in HIV viral load following depletion of these cells (**Figure 4E**). This observation indicated that anti-HIV activity was determined by the level of myeloid cells present. Based on these data, we concluded that myeloid cells are a significant determinant in controlling HIV replication in HIV-infected humanized mice.

**Figure 5:**
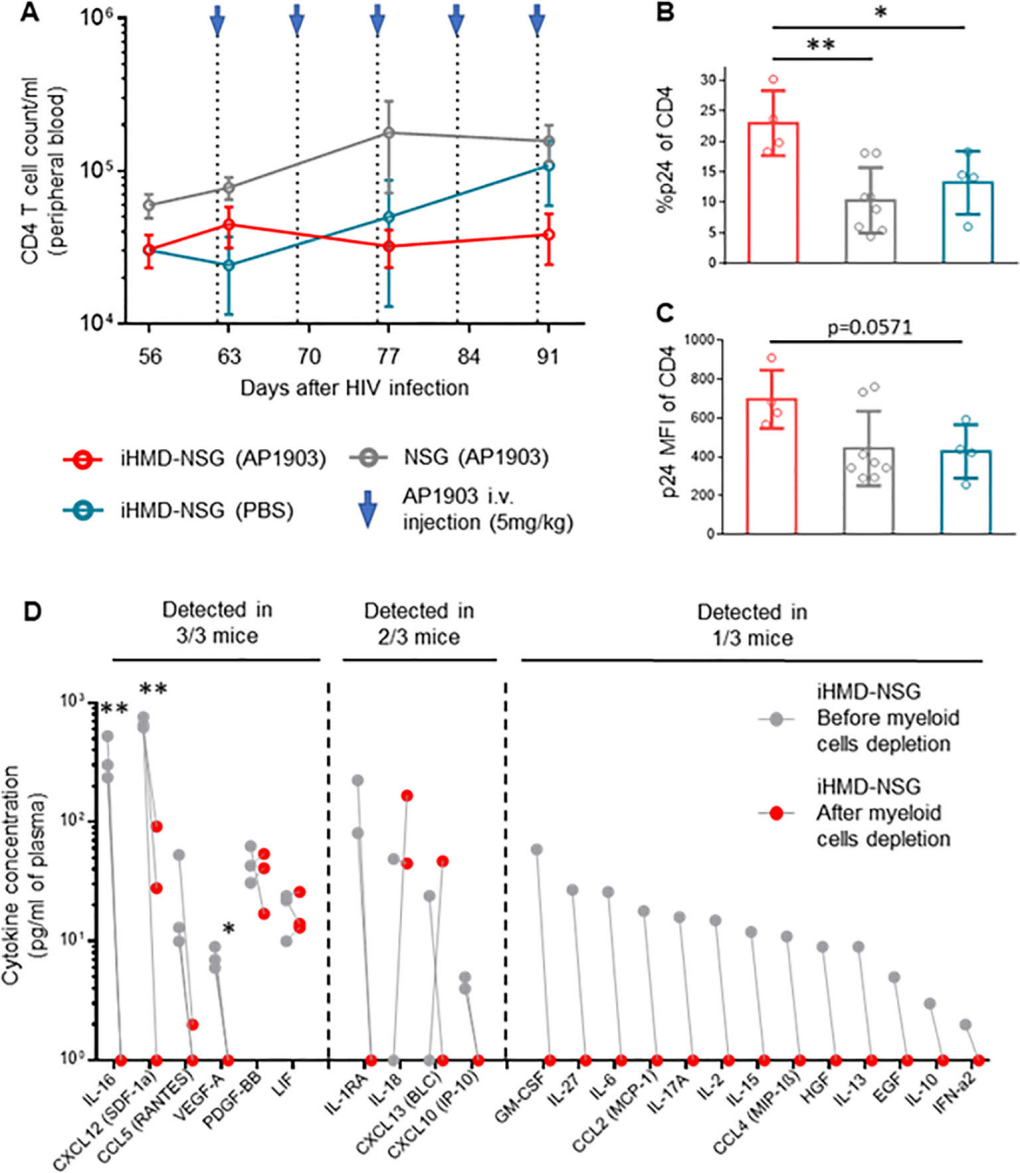
Depletion of myeloid cells results in increase of p24 expression in CD4+ T cells and downregulation of cytokines/chemokines. Hu NSG mice were treated with AP1903 (5mg/kg) (n=15), iHMD-NSG were treated with either AP1903 (5mg/kg) (n=13) or PBS (n=4) weekly, for 5 weeks. All mice were infected with YU-2. **(A)** CD4+ T cells count over time in the three different groups, calculated by using cell population frequencies, obtained by flow cytometry, on total peripheral blood cells processed per sample (Mean and standard error of the mean). **(B, C)** p24 intracellular expression in CD4+ T cells, evaluated via flow cytometry in bone marrow of iHMD NSG AP1903 treated mice (n=4), iHMD NSG mock treated mice (n=4), NSG AP1903 treated mice (n=8) (Mean and standard deviation). **(D)** Luminex multiplex assay for cytokine analysis, performed in plasma samples of 3 AP1903-treated iHMD NSG mice, before and after treatment. Statistic by Two-way ANOVA **(A)**, Mann-Whitney test **(B, C)**, two-tailed Student’s t-test **(D)** p-value: *<0.05; **<0.01; ***<0.001; ****<0.0001.

Longitudinal analysis showed no expansion of non-myeloid populations upon myeloid cell depletion (**Figure 5A**, **Supplementary Figure S10B**). However, we did not quantify the number of effector and central memory CD4+ T cells, which are the main target cells of productive HIV infection, and therefore we may have missed their expansion by only counting the total amount of CD4+ T cells. In any case, we detected higher intracellular HIV p24 antigen expression in CD4+ T cells from bone marrow of the AP1903-treated iHMD-NSG compared to the control groups (**Figures 5B, C**). Furthermore, we performed a cytokine analysis using plasma of iHMD-NSG mice obtained before and after myeloid cell depletion (**Figure 5D**). We observed downregulation of 19 cytokines after treatment with AP1903. Among these, chemokine receptor ligands and pro-inflammatory cytokines including CXCL12, CCL5, CCL4, IL-16, IL1RA, IL-27, IL-2, IL-10 have known anti-HIV effects (52–54). Taken together, these results suggest macrophages restrict HIV replication via secretion of soluble factors limiting viral production in CD4+ T cells.

### 3.5 Pro-inflammatory macrophages exert superior HIV inhibition *ex vivo* in a cell-to-cell independent manner

**Figure 6:**
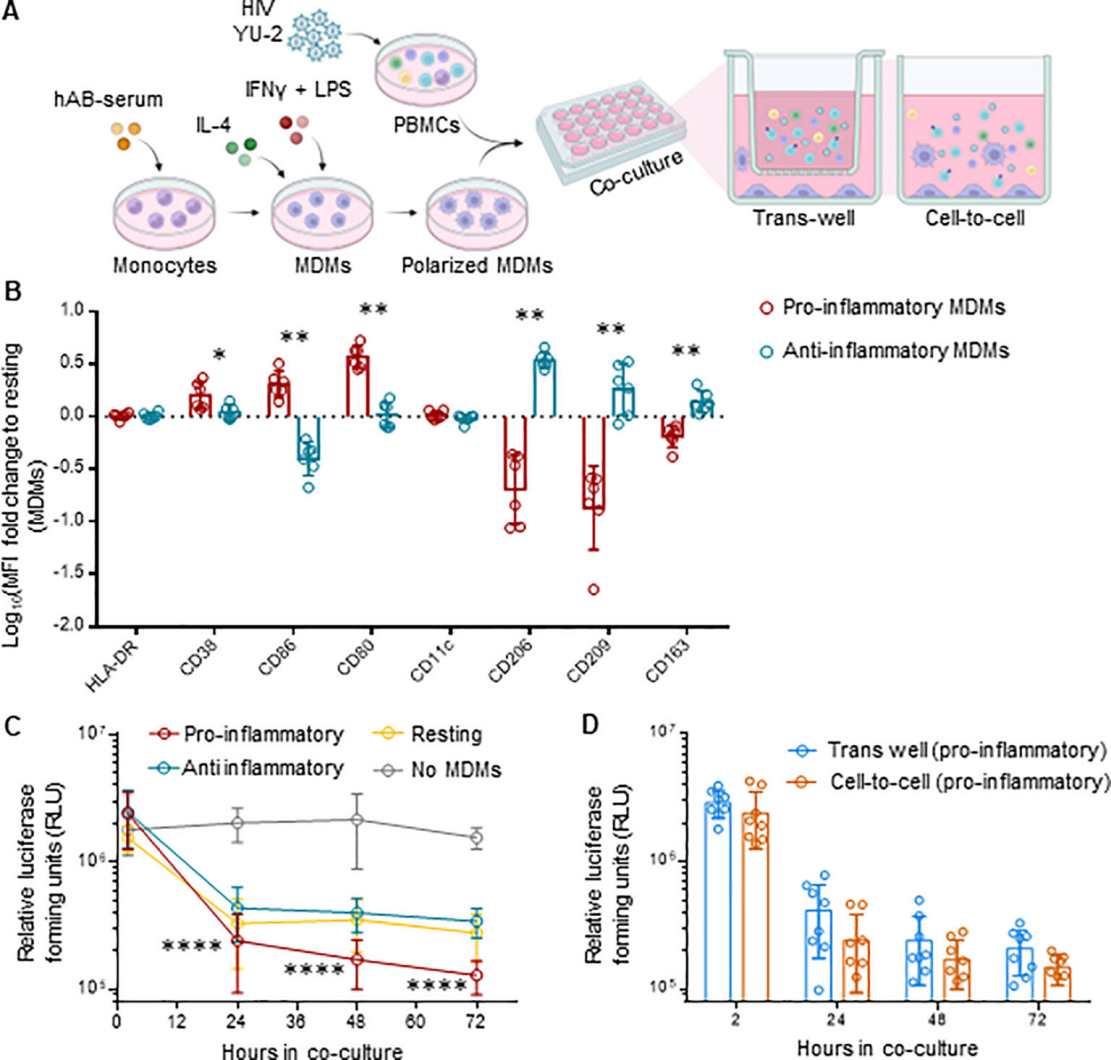
MDMs inhibit HIV replication in ex vivo co-cultures with HIV-infected PBMCs. **(A)** Schematics of experimental procedure, BioRender.com/u59h395 CD14+ monocytes were differentiated in MDMs for 6 days and polarized in either pro- (M1), anti-inflammatory (M2) or kept as resting (m0) MDMs. Differently polarized MDMs were co-cultured at a 1:1 ratio with autologous activated PBMCs infected with HIV NL4.3 2½ days ago. Co-cultures were either in direct contact (cell-to-cell) or separated by a trans-well (1 μm pore size). HIV infected PMBCs were also kept in monoculture as control. Culture supernatant was harvested at 2, 24, 48, 72 hours after co-culture setup for evaluation of HIV replication for each culture condition. **(B)** Flow cytometric analysis of polarized MDMs at the end of the experiment, i.e., 72 hours of co-cultivation, including pro-inflammatory (HLA-DR, CD38, CD86, CD80, CD11c) (n=6) and anti-inflammatory (HLA-DR, CD206, CD209, CD163) (n=6) myeloid surface markers. Displayed as logarithmic fold change to MFI of resting MDMs (Mean and standard deviation). **(C)** Viral replication for every cell-to-cell co-culture condition (n=8 per condition measured via TZM-bl luciferase assay at 2, 24, 48 and 72 hours post co-culture setup (Mean and standard deviation). **(D)** Viral replication of trans-well (n=8) and cell-to-cell (n=8) co-cultures for pro-inflammatory MDMs-HIV infected PMBCs, measured via TZM-bl luciferase assay at 2, 24, 48 and 72 hours post co-culture setup (Mean and standard deviation). Statistic by and Mann-Whitney **(B)**; two-way ANOVA **(C, D)**. p-value: *<0.05; **<0.01; ****<0.0001.

To validate our *in vivo* observations, we explored whether human macrophages exert control of HIV replication on *ex vivo* infected PBMCs. Therefore, we co-cultured *ex vivo* HIV-infected PBMCs with autologous M1 (pro-inflammatory), M2 (anti-inflammatory) polarized monocyte-derived macrophages (MDMs) or M0 (resting) MDMs, either directly (cell-to-cell) or separated by a trans-well (**Figure 6A**). The pore size of 1 µm of the trans-wells blocks cell passage while allowing free exchange of molecules (55).We infected PBMCs with the CXCR4-tropic HIV strain NL4-3, which does not productively infect macrophages. The use of this virus allowed us to examine the effects of macrophages on HIV replication in PBMCs uncoupled from potential productive HIV infection of macrophages – macrophages serving here rather as bystander cells. Polarization resulted in upregulation of CD38, CD86 and CD80 markers for pro-inflammatory MDMs, and CD206, CD209 and CD163 for anti-inflammatory MDMs, when compared to resting MDMs (**Figure 6B**, **Supplementary Figure S11A**). We found that MDMs, most notably the pro-inflammatory MDMs, inhibited HIV replication over time (**Figure 6C**, **Supplementary Figure S12**). Intriguingly, the anti-HIV effect persisted when the MDMs were physically separated by the trans-wells from the HIV-infected PBMCs (**Figure 6D**, **Supplementary Figures S11B, C**), which suggests a molecule-dependent rather than contact-dependent effect. Cytokine analysis resulted in a comparable profile to what we observed in iHMD-NSG mice (**Supplementary Figure S13**), with increased levels of anti-HIV soluble factors in the presence of pro-inflammatory MDMs. Furthermore, to roll out potential anti-HIV effects of other effector cells present in the co-culture, we depleted either NK cells or CD8+ T cells in both HIV-infected monocultures and co-cultures with pro-inflammatory MDMs (**Supplementary Figure S14A**). In all instances, no significant difference was observed when effector cells were depleted, strengthening MDMs’ anti-HIV effect. Overall, these *ex vivo* data are in line with the *in vivo* results, suggesting that pro-inflammatory macrophages have an HIV-inhibitory effect.

### 3.6 MDMs upregulate transcription of antiviral genes while downregulating DNA replication pathways in HIV-infected co-cultures

**Figure 7:**
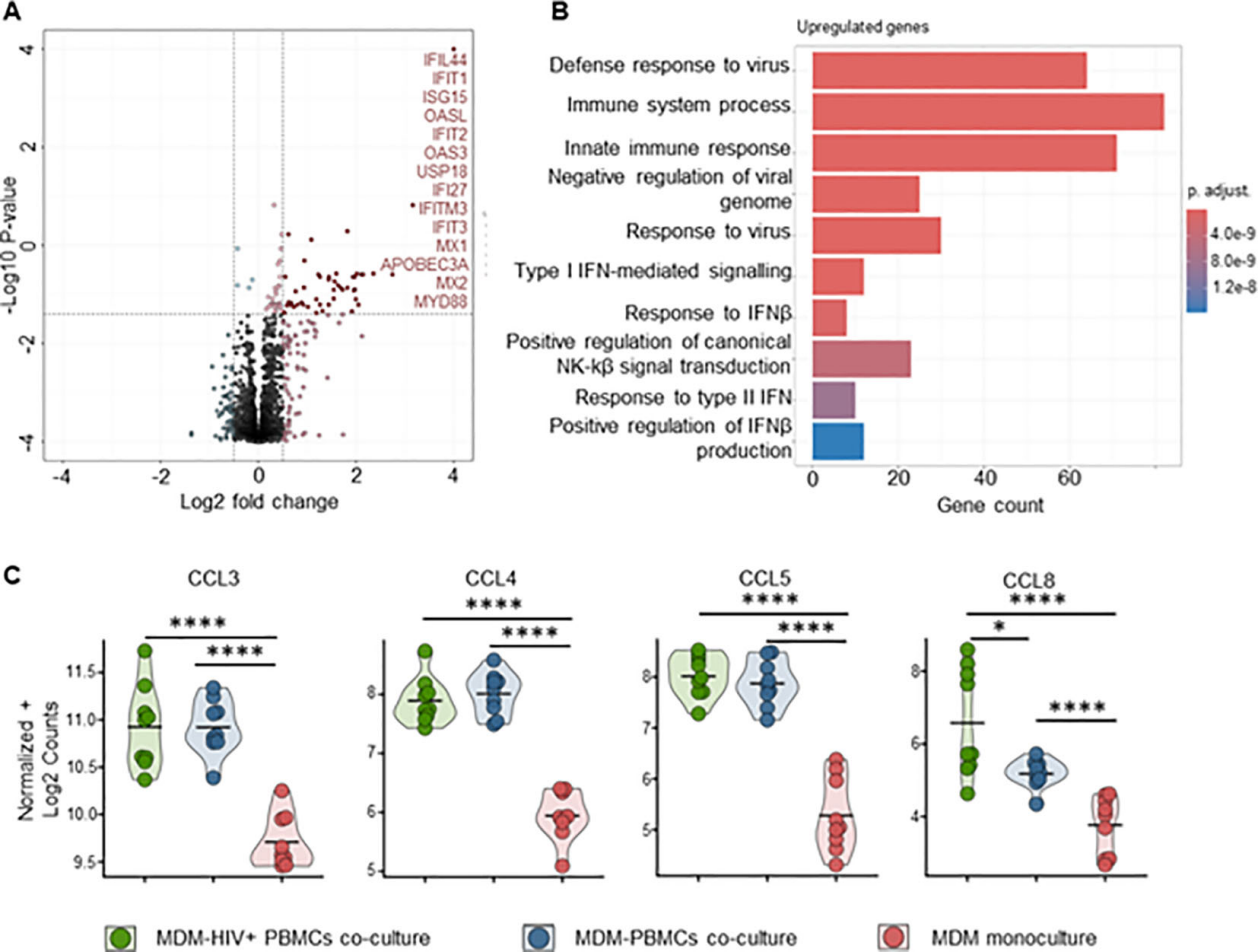
Pro-inflammatory (M1-polarized) MDMs bulk RNA sequencing shows increased antiviral response in the presence of HIV infected PBMCs **(A)** Volcano plot of differentially expressed genes in MDMs-PBMCs co-culture vs HIV+ MDMs-PBMCs co-culture (72h timepoint). Significant upregulated threshold 0.5 ≤ Log2 fold change ≥ 0.5; P-value ≤ 0.01. Red dots indicate significantly upregulated genes, annotated on the side. **(B)** Hypergeometric ORA test of upregulated biological processes of MDMs-PBMCs co-culture vs MDMs-HIV+ PBMCs co-culture comparison. **(C)** Violin plots of expression levels of HIV co-receptors ligands in MDMs in the 3 different culture conditions analyzed (Mean, n=9 per condition). Statistic by DESeq2 (see materials & methods section). FDR-adjusted p-value: *<0.05; ****<0.0001.

**Figure 8:**
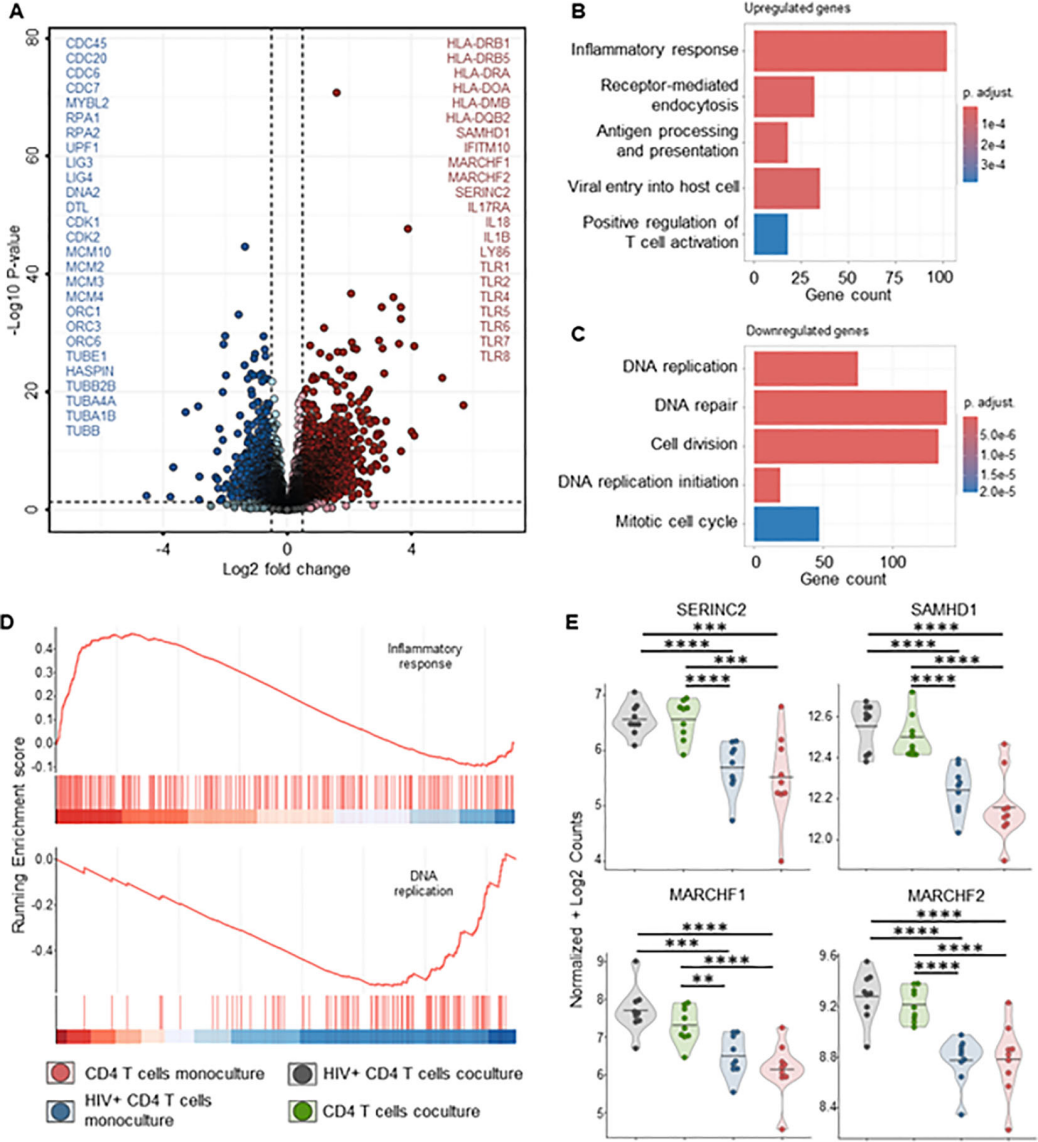
CD4+ T cells bulk RNA sequencing of HIV infected co-cultures of *ex vivo* HIV-infected PBMCs with macrophages shows increased T cell activation and decreased DNA replication in the presence of MDMs. **(A)** Volcano plot of differentially expressed genes in sorted CD4+ T cells of HIV infected PBMCs-MDMs co-cultures vs HIV infected PBMCs monoculture (72h time point). Significant upregulated threshold 0.5 ≤ Log2 fold change ≥ 0.5; P-value ≤ 0.01. Red dots indicate significantly upregulated genes in HIV infected PBMCs-MDMs co-cultures. Blue dots indicate significantly downregulated genes in HIV infected PBMCs-MDMs co-cultures. **(B)** Hypergeometric ORA test of upregulated biological processes of HIV infected PBMCs-MDMs co-cultures vs HIV infected PBMCs monoculture co-culture comparison. **(C)** Hypergeometric ORA test of downregulated biological processes of HIV infected PBMCs-MDMs co-cultures vs HIV infected PBMCs monoculture co-culture comparison. **(D)** GSEA showing significant enrichment of inflammatory response and downregulation of DNA replication in CD4+ T cells of HIV infected PBMCs-MDMs co-cultures vs HIV infected PBMCs monoculture. **(E)** Violin plots of expression levels of HIV restriction factors in MDMs in the 4 different culture conditions analyzed (Mean, n= 9 per condition). Statistic by DESeq2 (see materials & methods section). FDR-adjusted p-value: *<0.05; **<0.01; ***<0.001; ****<0.0001.

To better understand the mechanisms behind MDM’s control of HIV replication, we performed bulk RNA sequencing on both MDMs and CD4+ T-cells from mono- and co-cultures maintained for 72 hours, ± HIV infection. Transcriptome analysis of MDMs from HIV-infected co-cultures showed a significant fold increase of 48 genes compared to uninfected control, mainly ISGs (**Figure 7A**, **Supplementary Table S2**). Over-representation analysis (ORA) of these genes identified upregulated biological processes involved in defense response to the virus, immune system process and innate immune response (**Figures 7A, B**, **Supplementary Table S4**). We observed upregulation of HIV co-receptor ligands independently of HIV infection, i.e., significant increases in CCL3, CCL4, CCL5 and CCL8 (**Figure 7C**, **Supplementary Table S2**). Transcriptome analysis of sorted CD4+ T cells from either HIV-infected co-cultures or monocultures showed a consistent clustering of differently expressed features (**Supplementary Figures S15A, B**). The addition of autologous MDMs to HIV-infected PBMCs induced upregulation of transcripts involved in immune defense mechanisms in CD4+ T cells, including inflammatory response, antigen processing and T cell activation (**Figures 8A, B, D**). At the same time, we observed marked downregulation of DNA replication, DNA repair, cell division and mitosis (**Figures 8A, C, D**). Additionally, gene set enrichment analysis (GSEA) showed downregulation of mRNA splicing and rRNA production (**Supplementary Figure S15D**, **Supplementary Table S7**). Interestingly, among the upregulated genes in CD4+ T cells, we identified known HIV restriction factors, including SERINC2, SAMHD1, MARCHF1 and MARCHF2 (**Figure 8E**, **Supplementary Table S3**) (56). However, upregulation of these restriction factors appears to be dependent on the presence of MDMs but independent of HIV. Taken together, these results indicate that, during HIV infection, the presence of MDMs upregulates antiviral response while downregulating DNA replication in CD4+ T cells, leading to restriction of viral replication.

## 4 Discussion

Studies of macrophages in HIV infection so far have primarily focused on the contribution of these cells to the viral reservoir. In this study, we showed that macrophages have an anti-HIV effect both *in vivo* and *ex vivo*.

We first observed clear phenotypical differences between myeloid cells in HIV-infected and uninfected humanized mice. Upregulation of pro- and anti-inflammatory markers at different stages of infection clearly demonstrated macrophages’ ability to respond to HIV infection and is congruent with phenotypes observed in HIV-infected patients (23). In fact, we found an increased number of macrophages expressing pro-inflammatory markers during early HIV infection in multiple organs. This effect was further increased at the late infection time-point, indicating a constant activation of pro-inflammatory pathways, most likely due to the system’s inability to clear the infection. Different studies on cytokine profiles during HIV infection showed high levels of pro-inflammatory cytokines during acute HIV infection (57, 58). In contrast, an increase in anti-inflammatory cytokine production was observed during chronic infection stages (59, 60), which aligns with our findings of a significant increase of macrophages expressing anti-inflammatory markers at a late infection time point. Our results on myeloid cell phenotyping confirmed plasticity and functionality of macrophages in humanized NSG mice.

By depleting these mononuclear phagocytes, we aimed to understand the specific role of these cells during HIV infection. While knowing that clodronate kills mononuclear cells across species (48), we were intrigued to see a high increase in HIV RNA copy numbers in huNSG treated with clodronate. To note, murine mononuclear phagocytes in humanized mice, despite their defective nature (61), still maintain some functionality (62). In fact, murine macrophages may still phagocytose HIV-infected cells or engulf free virions (63). Furthermore, murine macrophage depletion in immunocompetent mice results in splenomegaly with extensive hematopoiesis (64), which we observed in clodronate-treated mice. Specifically, clodronate-treated mice displayed a significantly higher count of CD4+ T cells in both spleen and liver. Finally, clodronate-associated toxicity may result in T-cell activation, which in turn promotes HIV- replication. Thus, it is rather impossible to draw any firm conclusions on the role of human macrophages from these data.

To explore the role of human mononuclear phagocytes in HIV infection, we needed a hu mouse model that would allow for their specific depletion without any off-target effect. The iHMD model, based on the transplantation of genetically modified HSPCs harboring a myeloid-specific iCasp9, achieved such results. The lower human engraftment level in iHMD-NSG mice compared to plain huNSG mice is most likely a result of the *ex vivo* manipulation of HSPCs (65, 66). It is important to note that the iHMD-NSG and huNSG mice were comparable in leukocyte distribution. GFP expression, driven by the myeloid-specific promoter, was highest in human myeloid cells, low in NK cells and negligible in T and B cells. Notably, a small fraction of NK cells originate from myeloid progenitor cells, explaining their low GFP expression (67). The less than 100% GFP expression in myeloid cells is likely a result of stochastic epigenetic silencing of the transgene during hematopoiesis (68). In fact, genes introduced by lentiviral transduction randomly integrate into active transcription units of the host cell genome (69) and depending on the integration site, epigenetic silencing may occur. Following AP1903 treatment, we observed a depletion of >95% of GFP+ myeloid cells, similar to what was reported by Stasi et al. (49). The total number of myeloid cells decreased by >90%, which suggests either an extremely high sensitivity of the iCasp9 system or an underestimation of the iCasp9 transgene detected. The latter can be explained by the polycistronic structure of the vector, where a 2A self-cleaving peptide separates the iCasp9 gene and GFP. Liu et al. showed that protein expression decreases the further away from the promoter (70). We also achieved the elimination of GFP+ myeloid cells over time by weekly treatment with AP1903. Thus, the iHMD mouse model permits selective human myeloid cell depletion without off-target effects, while maintaining human engraftment.

AP1903-treatment in HIV-infected iHMD-NSG mice resulted in a rapid and significant increase in HIV load, which persisted over time. Notably, human macrophages in the spleen and bone marrow were HIV infected in huNSG mice (**Supplementary Figure S16**). However, the half-life of free HIV virions in the peripheral blood is estimated to be about 48h (71, 72). Therefore, the constant and stable increase in viral load cannot be explained by the release of free virions from productively HIV-infected apoptotic macrophages. Although we did not observe CD4+ T cell expansion after macrophage depletion as a cause of the increase in HIV replication, we cannot completely rule it out due to a possible insufficient sample size. Likewise, we cannot rule out that we missed a subtle expansion of effector and/or central memory CD4+ T cells, the main HIV producer cells, since we only counted the number of total CD4+ T cells. In any case, we found a higher frequency of HIV-infected CD4+ T-cells as well as higher intracellular HIV p24 expression, which could explain the increased HIV replication. Interestingly, we observed decreases in several cytokines and chemokines, which were mostly undetectable in iHMD-NSG mice after the depletion of human myeloid cells. Chemokines such as CCL5, CCL4 and CXCL12 act as HIV entry inhibitors by binding to HIV co-receptors (52, 53). Pro-inflammatory cytokines, IL-16, IL1RA, IL-27, IL-2, IL-10 and IFNα, inhibit HIV replication via different mechanisms (54). On the other hand, cytokines, such as IL-6, IL-15 and GM-CSF, were shown to favor HIV replication by inducing expansion of HIV-infected cells (54). Ultimately, the net effect of all the chemokine/cytokine changes will likely shape the anti-HIV activity of macrophages. Our data in iHMD-NSG mice suggest that chemokines/cytokines mediate, at least in part, the HIV inhibitory effects of macrophages. To note, this is the first study ever to demonstrate an anti-HIV-specific effect of human macrophages *in vivo* through their selective degradation.

To better characterize these mechanisms, we further investigated human macrophages’ anti-HIV activity by co-culturing MDMs with *ex vivo* HIV-infected PBMCs. Here, we used the CXCR4-tropic strain NL4-3 which does not result in productive infection of macrophages (73). This allowed us to investigate whether macrophages exert an HIV inhibiting effect in PBMCs without being infected themselves. In fact, the addition of MDMs, particularly pro-inflammatory MDMs, led to a significant reduction in HIV replication even in NL4-3 infected co-cultures. Furthermore, finding no differences in HIV replication, regardless of whether the co-cultures were in direct contact or separated by trans-wells argues that soluble factors are at the basis of macrophage’s anti-HIV activity. Cytokine analysis revealed expression patterns comparable to our *in vivo* analysis, with higher anti-HIV soluble factor concentrations in the presence of pro-inflammatory MDMs. Furthermore, this HIV inhibiting effect requires no productive HIV infection of macrophages. Similarly, other research groups have observed a macrophage-directed anti-viral response via the upregulation of cytokines and chemokines in other virus infections, such as HBV and HCV, Zika virus and Dengue (74–77).

Transcriptomic analysis on MDMs strengthen our observations at a protein level that macrophages are producers of anti-HIV cytokines and chemokines. In fact, MDMs co-cultured with PBMCs, undergo a strong immune activation, together with upregulation of chemokines and cytokines (**Supplementary Figure S17A**). In particular, we identified the chemokine receptor ligands CCL3, CCL4, CCL5 and CCL8. All of which bind to HIV co-receptor CCR5 and suppress HIV replication *in vitro* (53, 78–81). In the presence of HIV-infected cells, MDMs showed a transcription profile dominated by anti-viral ISGs (82–85), many of which are known as HIV restriction factors. This signature hints to IFN-α as main trigger of macrophage activation. Macrophages changed the transcriptomic profile of CD4+ T from HIV-infected co-cultures as well. In addition to immune activation, we observed a marked downregulation of genes involved in biological processes ranging from DNA replication to protein production and cell division. Consistent with our data, macrophages were recently described to reduce HIV expression in memory CD4+ T cells *in vitro* by modulating cell activation (86). The downregulation of DNA transcription and RNA translation in HIV-infected CD4+ T cells can explain the decrease in HIV production from HIV-infected cells. Notably, proliferating CD4+ T cells are the most susceptible one to HIV replication (87). Downregulation of cell division and replication is likely to result in a decrease of new HIV infection of T cells, further promoting the decrease in HIV production.

In this study, we investigated the role of macrophages as effector cells both *in vivo*, using a CCR5-tropic strain, and *ex vivo*, using a CXCR4-tropic strain. By employing HIV strains with different co-receptor selectivity, we introduced an additional variable, yet both the *in vivo* and *ex vivo* experiments produced consistent results. We believe that the observed inhibitory effects of macrophages on HIV are not related to HIV co-receptor selectivity, but this hypothesis needs to be formally tested in future studies. Notably, YU-2 is a primary HIV strain that was directly isolated from the brain and is well characterized for its ability to infect macrophages (88). Global mapping of the HIV-1 transcriptome reveals that both productively infected macrophages and bystander cells exhibit similar activation of the type-1 IFN signaling pathway, suggesting that these two cell populations were exposed to the same triggering signals (89). In contrast, the induction of susceptibility genes related to DNA reorganization required for viral integration and transcription appeared to be specific to productively infected macrophages. Data exploring the impact of CXCR4-tropic strains on macrophages are currently lacking. It is possible that CXCR4-tropic strains trigger the same sensing receptors in the endosomal compartment as CCR5-tropic strains, which is decoupled from productive HIV infection. A particularly intriguing study showed that IFN activation in plasmacytoid dendritic cells depends on the HIV Env–CD4 interaction and the targeting of HIV to early endosomes (90). That might be also the case for macrophages. However, with the current data, we cannot make any definitive statements regarding the detailed mechanisms underlying the anti-HIV effects we observed or to what extent CCR5-tropic HIV infections differ from CXCR4-tropic HIV infections in connection with the inhibitory effect of macrophages. Single-cell RNA sequencing of macrophages isolated from humanized mice infected with HIV strains having distinct co-receptor selectivity is necessary to address these questions. It should be noted that, in addition to the HIV envelope, other HIV genes may influence HIV replication in the various cellular compartments.

Other intriguing questions remain such as the role of mononuclear cells in cART treated subjects that can be addressed using this iHMD-NSG model, how long does it take to reconstitute the myeloid compartment when AP1903 is stopped, and what happens to the HIV replication in that situation and what happens if AP1903 is administered during acute infection vs chronic infection? When interpreting the results of this work, we must be aware of the limitations of the human immune system in humanized NSG mice (91, 92). We would like to emphasize that humanized mice are the best *in vivo* small animal model to study HIV pathogenesis. It is certainly a very accurate model to study the response of the innate immune system to the HIV challenge. The major shortcoming of humanized mice is the absence of an efficient immune system that of course may have a reciprocal impact on the innate immune response (93). We could imagine that the potential of myeloid cells could be even bigger on the HIV replication in the case of an adaptive immune system. Furthermore, great efforts have been made to improve the human immune system by providing human transgenes (94). It would be interesting to see to what extent a superior myeloid compartment as present for example in humanized MISTRG mice (95) would have resulted in a different outcome. We believe this will not be the case, but that remains speculation.

In summary, our study demonstrated a novel role of macrophages in restricting HIV infection during natural HIV infection. We showed that macrophages produce anti-HIV ISGs and chemokine-ligands, while inducing downregulation of DNA replication in CD4+ T cells which may be the origin of their HIV inhibitory activity. A more in-depth characterization of the anti-HIV effects of these cells could open up new possible immunotherapeutic approaches to HIV infection. While the current focus is primarily on dendritic cell immunotherapy, we believe in the overall HIV-inhibitory potential of myeloid cells coupled with their potential to generate an HIV-specific immune response that could ultimately lead to control of HIV without cART (96).

## Data Availability

The datasets presented in this study can be found in online repositories. The names of the repository/repositories and accession number(s) can be found below: GSE269285 (GEO).
