# Supplementary material for: Pro-inflammatory macrophages suppress HIV replication in humanized mice and ex vivo co-cultures
Source: Front Immunol. 2024 Nov 7;15:1439328. doi: 10.3389/fimmu.2024.1439328 (PMC11578737; doi:10.3389/fimmu.2024.1439328)
Supplement: Supplementary file 2 [file Table2.docx]

**Supplementary material:**


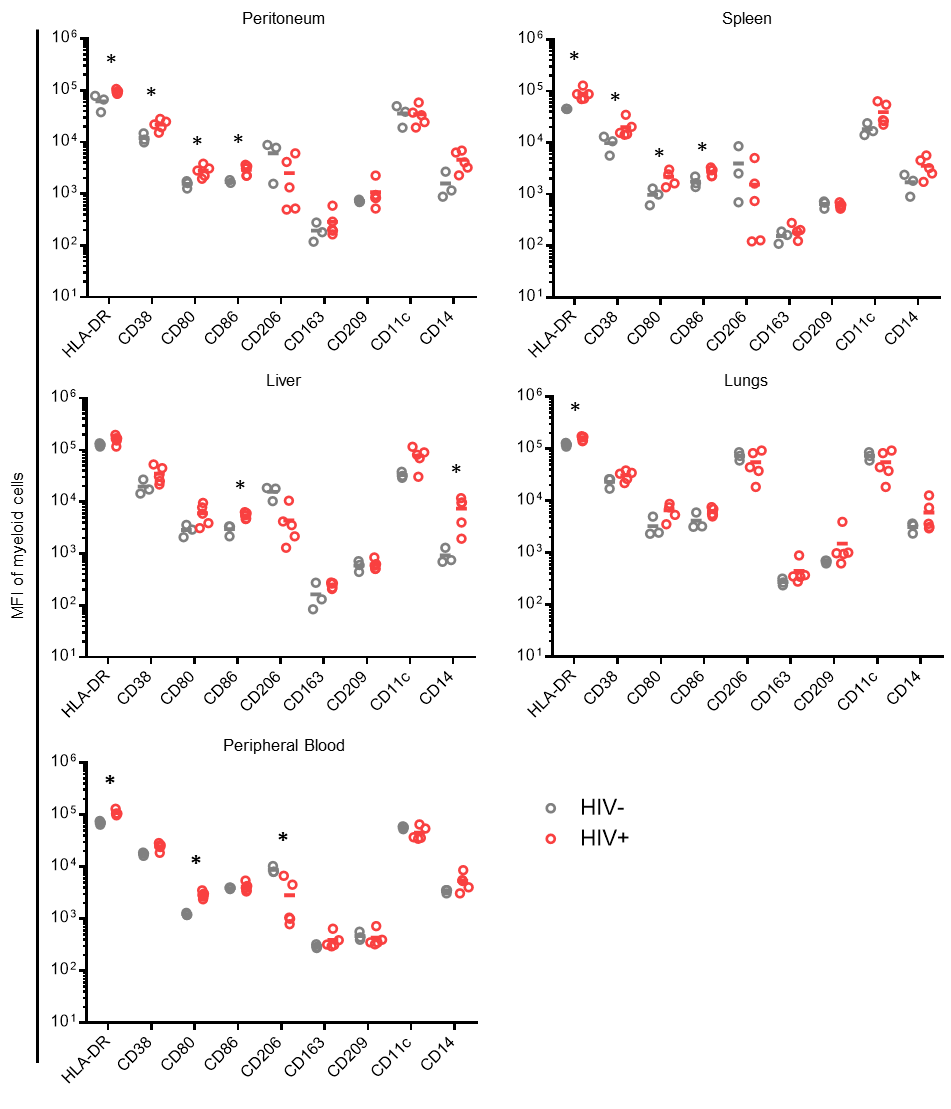


**Fig. S1 Myeloid surface markers expression during early HIV infection*.*** Mean fluorescence intensity (MFI) of human myeloid cells in peritoneum, spleen, liver, lungs and peripheral blood 4 weeks after HIV-1 (n=5) or mock infection (n=3) (Mean). Statistics by Mann-Whitney. p-value: *<0.05; **<0.01; ***<0.001; ****<0.0001


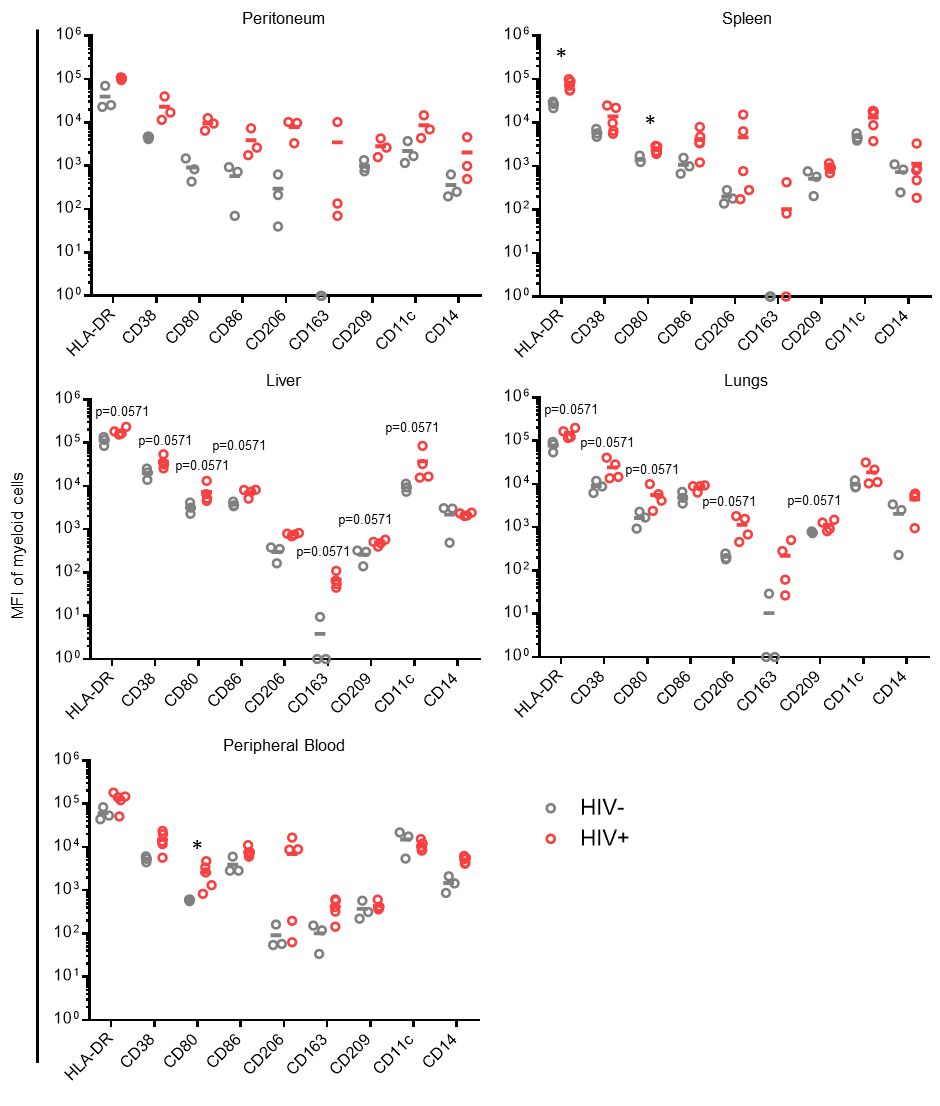


**Fig. S2 Myeloid surface markers expression during late HIV infection*.*** Mean fluorescence intensity of human myeloid cells in peritoneum, spleen, liver, lungs and peripheral blood, 12 weeks after either HIV-1 (n=5) or mock infection (n=3) (Mean). Statistics by Mann-Whitney. p-value: *<0.05; **<0.01; ***<0.001; ****<0.0001


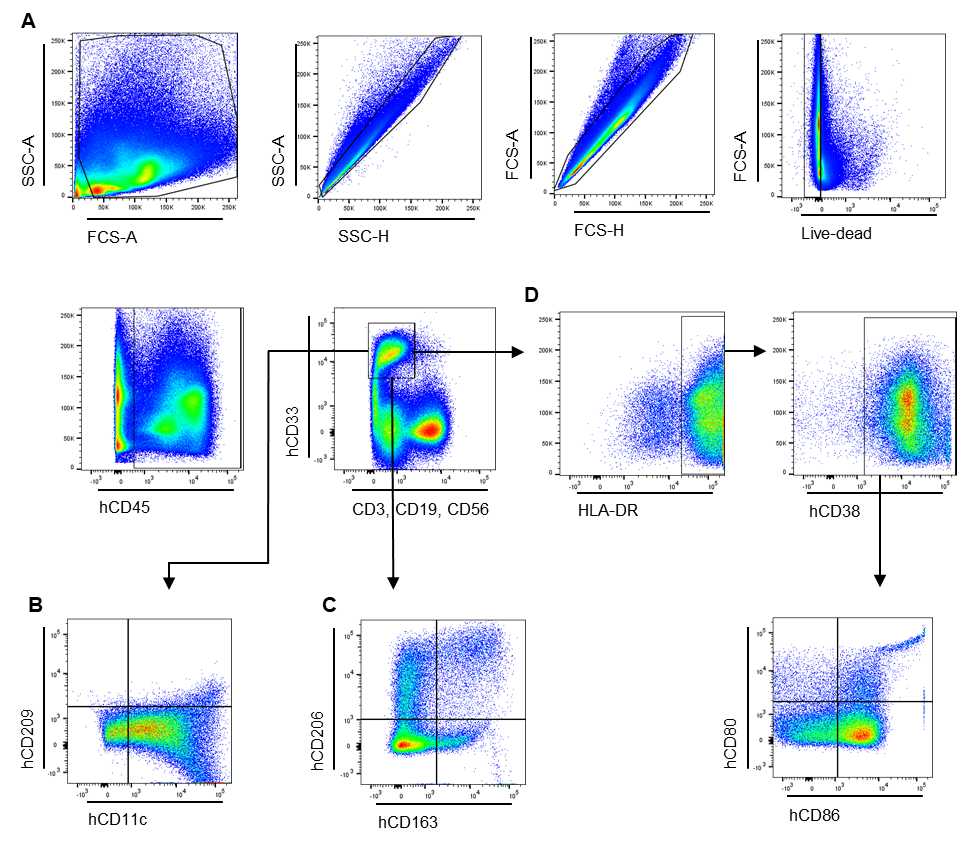


**Fig. S3 Gating strategy for flow cytometry detection of mononuclear phagocytes.** (**A**) gating of leukocytes to human myeloid cells (hCD45+, CD3-, CD19-, CD56-, CD33+). From myeloid cells (**B**) dendritic cells hCD209+, hCD11c+; (**C**) anti-inflammatory macrophages hCD206+, hCD1163+, (**D**) HLA-DR+, hCD38+, hCD80+, hCD86+.


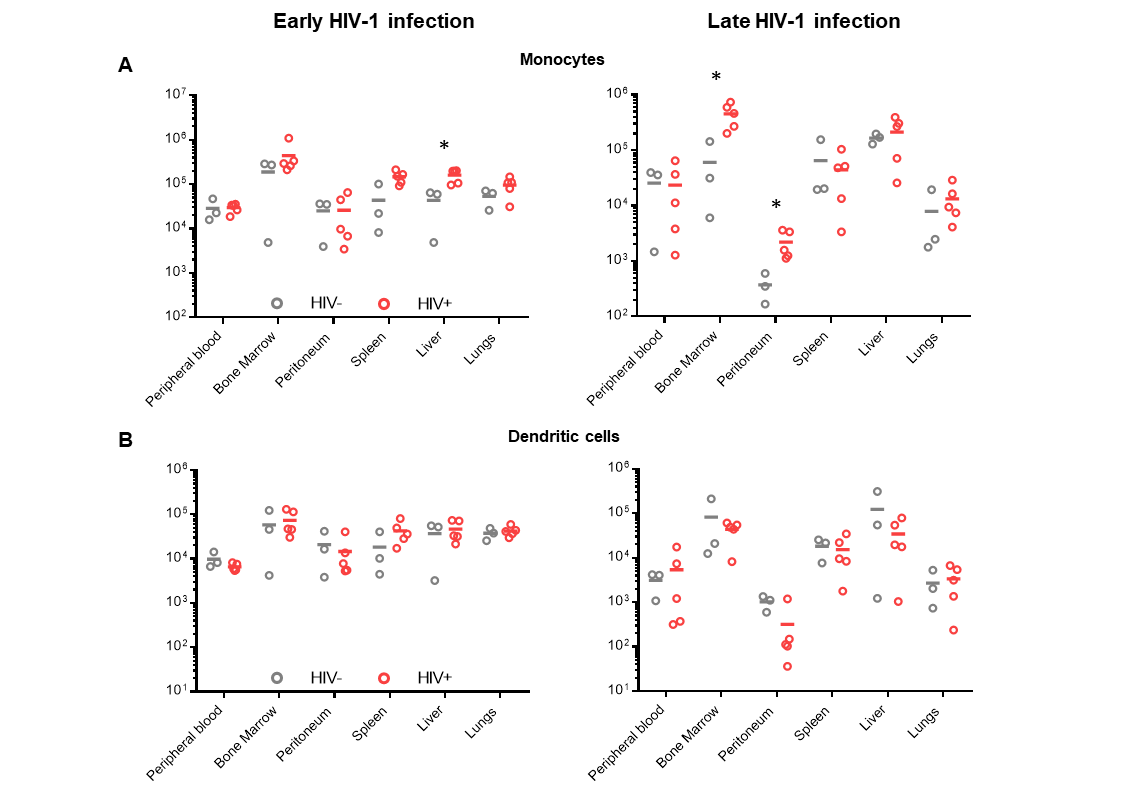


**Fig. S4. Mononuclear phagocytes count during early and late HIV infection** (**A**) Total count per organ of human monocytes (CD14+, HLA-DR+, CD3-, CD19-, CD56-), during early (4 weeks) and late (12 weeks) infection, in HIV infected (n=5) and uninfected (n=3) (Mean). (**B**) Total count per organ of human dendritic cells (CD209+, CD11c+, CD3-, CD19-, CD56-), during early (4 weeks) and late (12 weeks) infection, in HIV infected (n=5) and uninfected (n=3) (Mean). Cell counts were calculated by using cell population frequencies, obtained by flow cytometry, on total cells processed per sample. Statistic by Mann-Whitney. p-value: *<0.05; **<0.01; ***<0.001; ****<0.0001


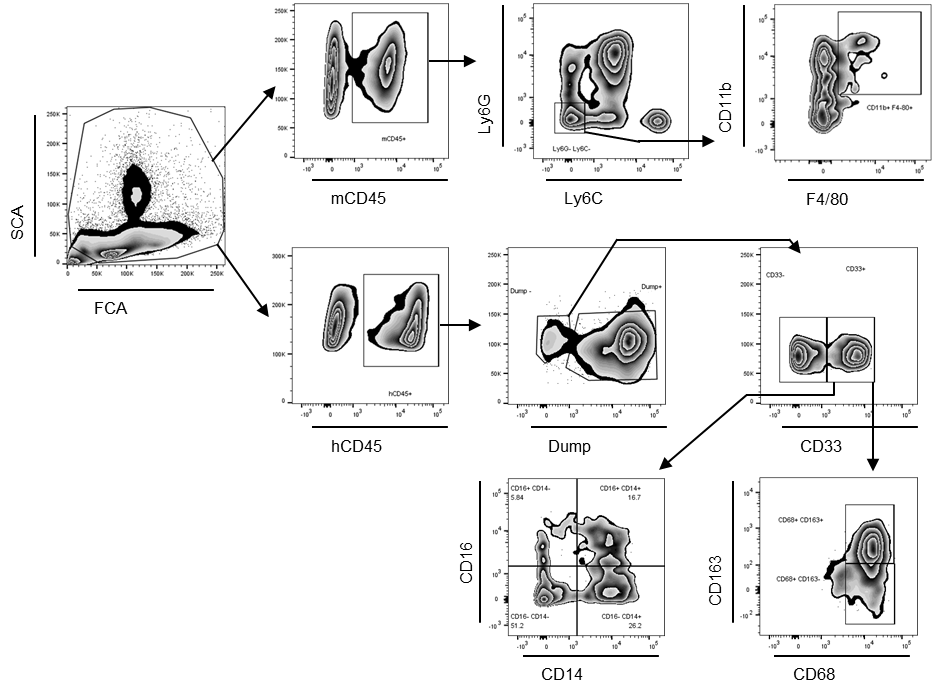


**Fig. S5 Gating strategy for identification of murine and human monocytes/macrophages** Gating strategy for identification of either murine monocytes/macrophages (m45+, ly6C-, ly6G-, CD11b+, F4/80+) or human monocyte macrophages (hCD45+, CD3-, CD19-, CD56-, CD33+, CD68+), via flow cytometry.


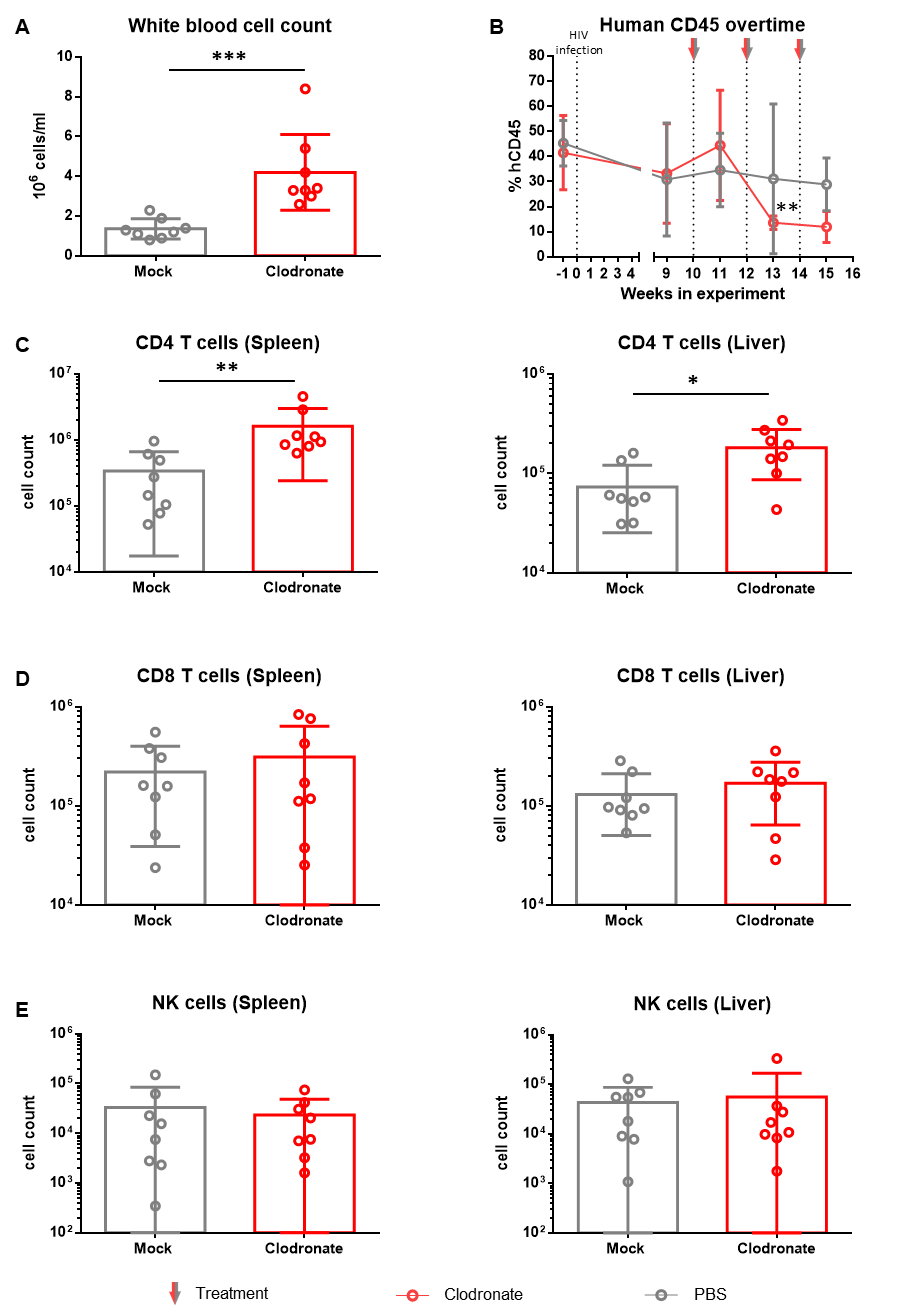


**Fig. S6 Human non-myeloid leukocyte populations distribution in PBS (mock) and clodronate treated HIV infected NSG mice.** (**A**) Total white blood cell count in peripheral blood at experimental endpoint. (**B**) Human CD45 frequency overtime in peripheral blood. (**C**) CD4+ T cells (hCD45+, CD3+, CD56-, CD4+, CD8-) count in spleen and liver at experimental endpoint. (**D**) CD8+ T cells (hCD45+, CD3+, CD56-, CD4-, CD8+) count in spleen and liver at experimental endpoint. (**E**) NK cells (hCD45+, CD3-, CD56+) count in spleen and liver at experimental endpoint. Statistics by Mann-Whitney. p-value: *<0.05; **<0.01; ***<0.001; ****<0.0001. Statistic by One-way ANOVA (**B**), Mann-Whitney test (**A** and **C** to **E**). P-value: *<0.05; **<0.01; ***<0.001; ****<0.0001


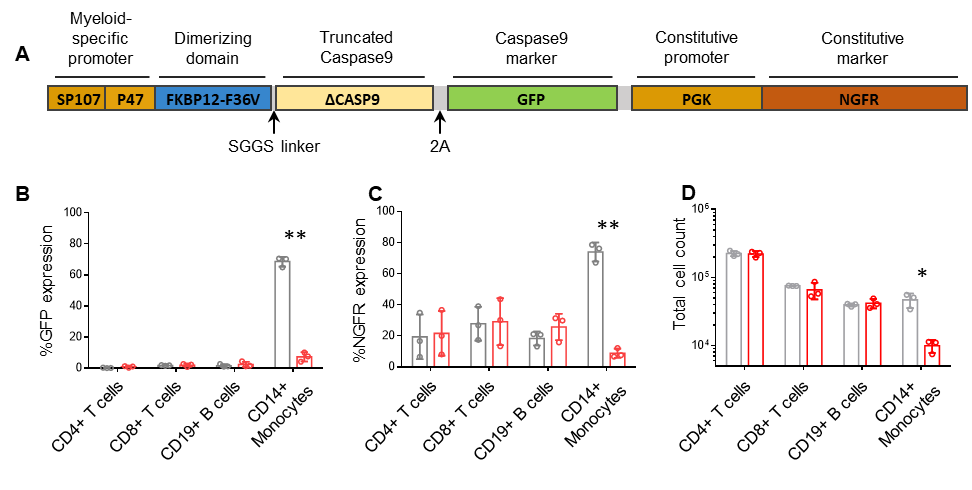


**Fig. S7 Generation and ex vivo testing of a myeloid specific suicide gene.** (**A**) Schematics of the lentiviral construct. Myeloid-specific synthetic promoter (SP107-p47), driving transcription of iCasp9, composed by the werdimerizing domain (FKBP12-F36V), the truncated caspase 9, green florescent protein (GFP) employed as iCasp9 expression maker. Human phosphoglycerate kinase 1 promoter (pGK) was inserted as constitutive promoter for CD34+ cell transduction, with a truncated version of the nerve growth factor (NGFR), as marker. (**B**-**D**) Flow cytometry analysis of PBMCs transduced with lentiviral particle carrying the construct in (**A**) (Mean and standard deviation). PBMCs were then treated either with AP1903 (10nM) (n=3) or DMSO (diluted 1:105 in PBS) as mock treatment (n=3), for 24 hours. Quantification of (**B**) GFP and (**C**) NGFR expression in monocytes (CD14+, CD3-), CD4+ T cells (CD3+, CD19-, CD4+, CD8-), CD8+ T cells (CD3+, CD19-, CD4-, CD8+) and B cells (CD3-, CD19+). (**D**) Cell counts for each cell type were calculated by using cell population frequencies, obtained by flow cytometry, on total cells processed per sample. Statistics by Mann-Whitney (**B**-**D**). p-value: *<0.05; **<0.01; ***<0.001; ****<0.0001


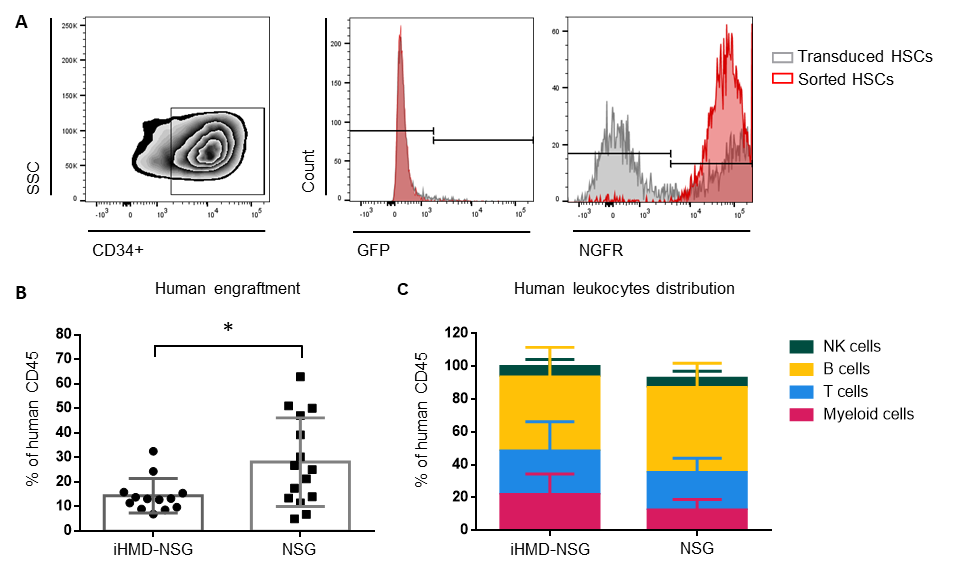


**Fig. S8 Generation of iHMD-NSG mice and engraftment check.** (**A**) CD34+ cells transduction and sorting of CD34+, NGFR+ cells. (**B**) Human engraftment that was checked at 16 weeks of age and (**C**) leukocytes distribution, analysed via flow cytometry in mice engrafted with genetically engineered HSPCs (iHMD-NSG, n=13) cells and mice engrafted with canonical HSPCs (NSG, n=15) (Mean and standard deviation). Statistic by Mann-Whitney. p-value: *<0.05; **<0.01; ***<0.001; ****<0.0001


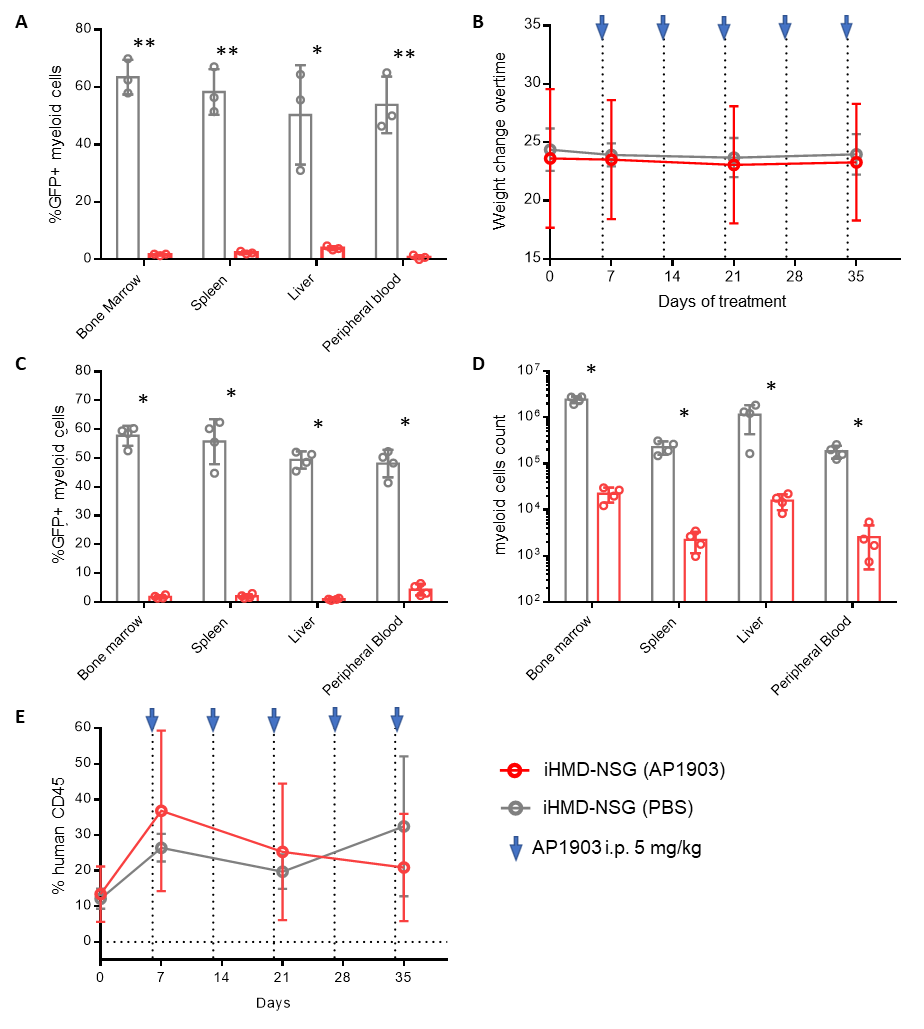


**Fig. S9 iHMD-NSG model characterization** (**A**) GFP expression in monocyte/macrophages of iHMD-NSG mice after 24hrs of either AP1903 (n=3) or PBS (n=3) single injection obtained via flow cytometry (Mean and standard deviation). (**B**) Weight change overtime over 5 weeks treatment period AP1903 (n=4) or PBS (n=4) (Mean and standard deviation). (**C**) GFP expression in myeloid cells (CD3-, CD19-, CD56-, CD33+) in organs after 5 weeks of treatment, obtained via flow cytometry (Mean and standard deviation). (**D**) Absolute myeloid cells numbers, calculated by using cell population frequencies, obtained by flow cytometry, on total cells processed per sample (Mean and standard deviation). (**E**) Percentage of human CD45+ expression overtime (day 7, 21, 35), evaluated in peripheral blood via flow cytometry (Mean and standard deviation). Statistic by Mann-Whitney (**A**, **C**, **D**); two-way ANOVA (**B**, **E**). p-value: *<0.05; **<0.01; ***<0.001; ****<0.0001


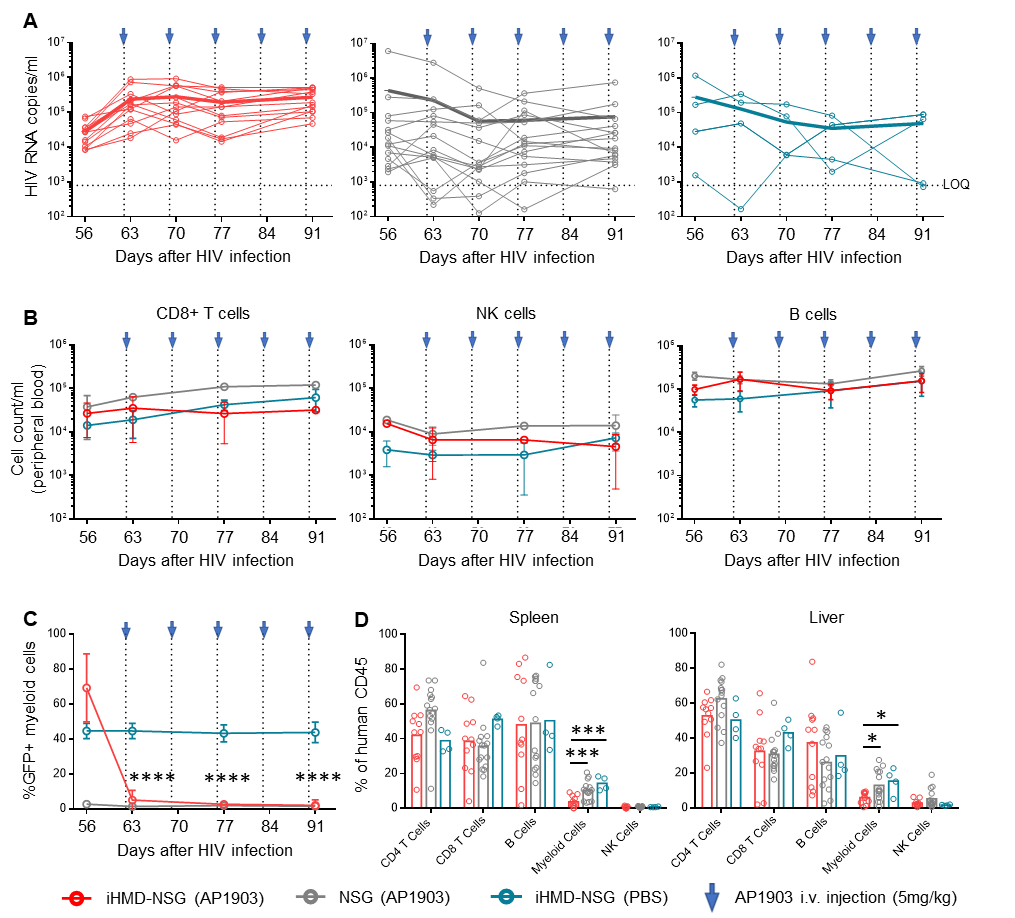


**Fig. S10 Effect of myeloid cell depletion on HIV viral load and leukocytes.** (**A**) Viral load overtime plotted as single mice per group (thick line represents mean per group, LOQ= limit of quantification). (**B**) Absolute count of multiple cell types per ml of peripheral blood over the course of treatment, calculated interpolating cell frequencies to samples cell counts (Mean and standard deviation). (**C**) GFP expression in myeloid cells overtime, measured via flow cytometry for all treatment groups: iHMD-NSG (AP1903) (n=13), NSG (AP1903) (n=15), iHMD-NSG (4). (Mean and standard deviation). (**D**) Leukocyte distribution in spleen and liver for all treatment groups at experimental endpoint (Mean). Statistic by two-way ANOVA (**B** and **C**); Mann-Whitney (**D**). p-value: *<0.05; **<0.01; ***<0.001; ****<0.0001


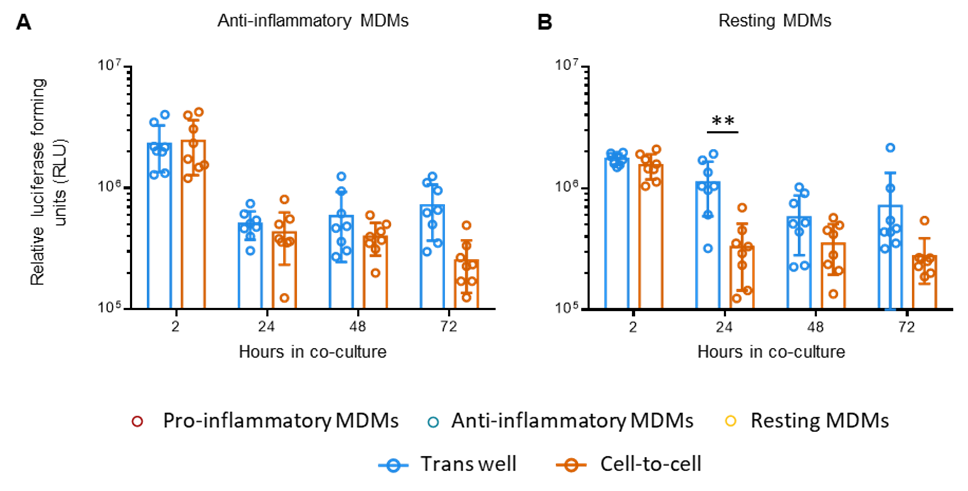


**Fig. S11** **Viral replication in anti-inflammatory and resting MDMs, trans well vs cell-to-cell** (**A**) Viral replication in *ex vivo* co-cultures with either anti-inflammatory (n=8) or resting MDMs (n=8) (**B**) cultured together or separated by trans wells (Mean and standard deviation). Viral replication was determined using the TZM-bl luciferase assay. Statistics by Mann-Whitney. p-value: *<0.05; **<0.01; ***<0.001; ****<0.0001.


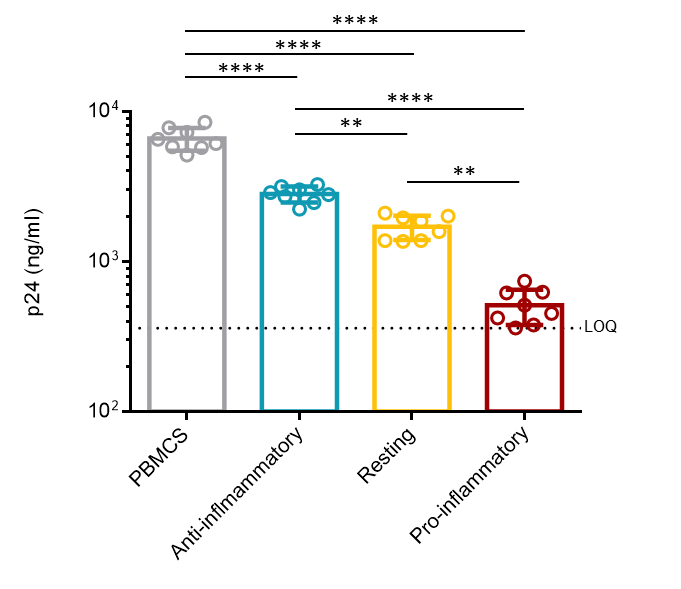


**Fig. S12** **HIV p24 ELISA at co-culture experimental endpoint (72h) of cell-to-cell co-cultures.** HIV p24 ELISA in HIV infected PBMCs either alone or co-cultured for 72 hours with either anti-inflammatory, resting or pro-inflammatory MDMs (n=8 per condition). For each condition, cells were cultured in direct contact. Statistics by Mann-Whitney. p-value: *<0.05; **<0.01; ***<0.001; ****<0.0001.


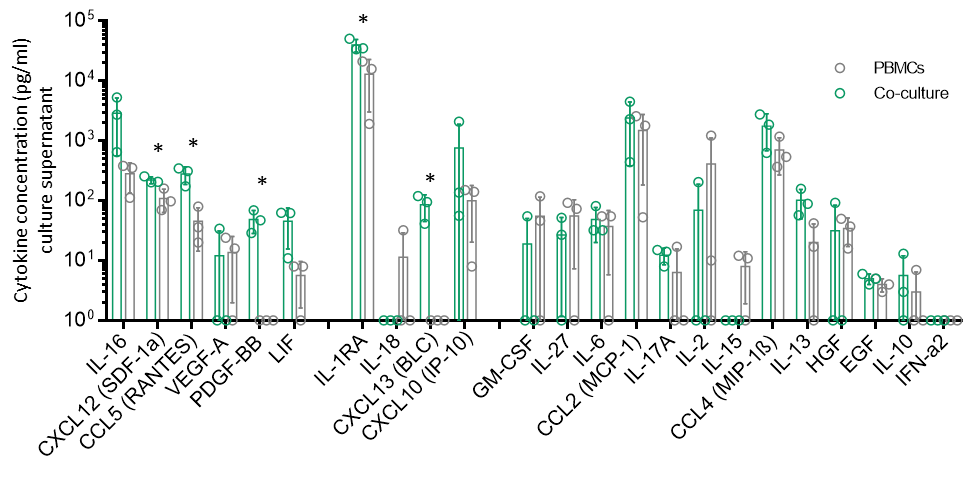


**Fig. S13 Cytokine evaluation in pro-inflammatory MDMs co-cultures vs HIV-infected PBMCs monocultures.** Luminex multiplex assay for cytokine analysis was performed in samples from either HIV-infected PBMCs (PBMCs) (n=3) or pro-inflammatory MDMs co-cultured with HIV-infected PBMCs (co-culture) in cell-to-cell fashion (n=3) (Mean±STD)~~.~~ We plotted here the cytokines we detected in iHMD-NSG (Fig. 5D). Each dot represents a sample. Statistic by Mann-Whitney test p-value: *<0.05; **<0.01; ***<0.001; ****<0.0001


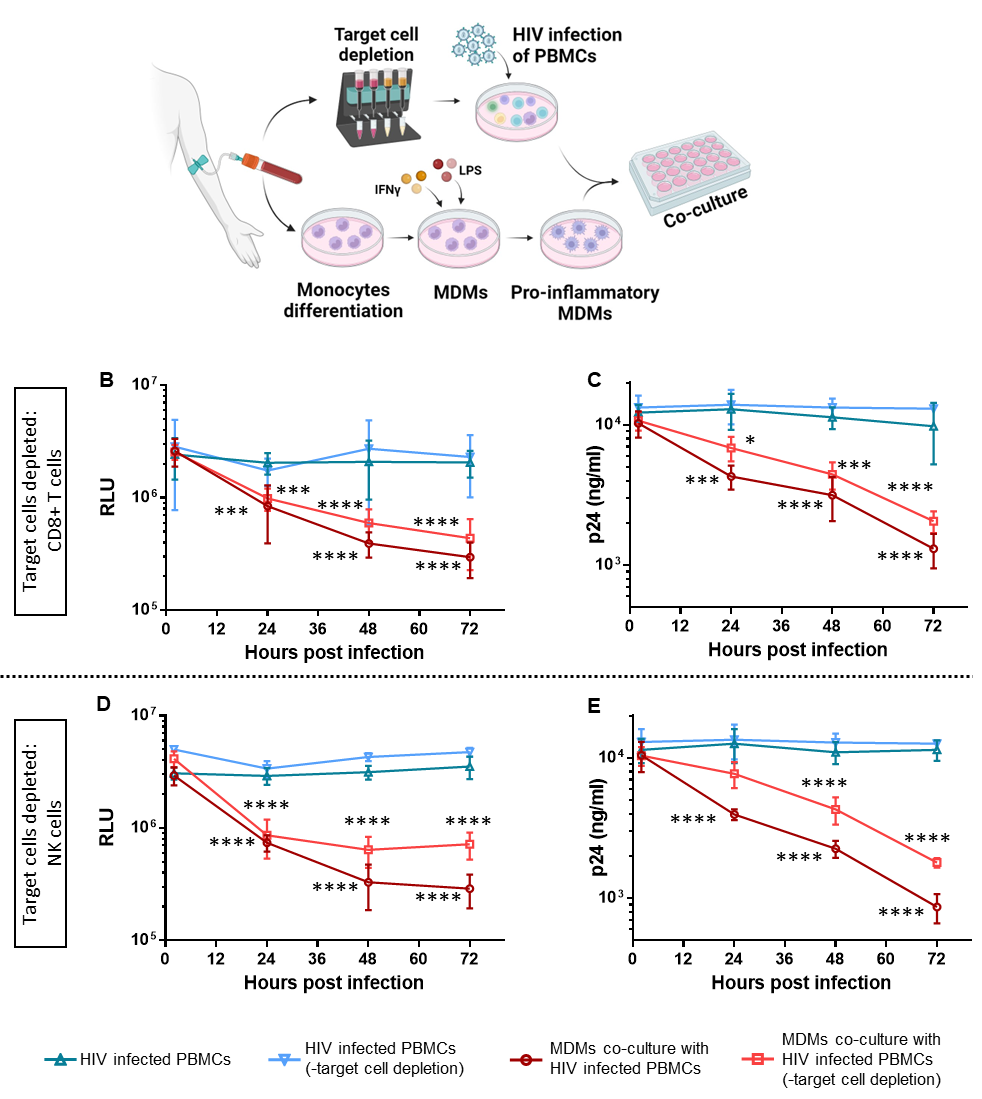


**Fig. S14 Targeted effector cell depletion to assess role of NK and CD8 T cells in co-culture system.** (**A**) Schematics of experimental procedure created with Biorender.com (**B, D**) viral replication overtime measured via TZM-bl luciferase assay at 2, 24, 48 and 72 hours post co-culture setup (Mean and standard deviation). (**C, E**) HIV p24 production overtime measured via ELISA at 2, 24, 48 and 72 hours post co-culture setup (Mean and standard deviation). Statistics by Two-way ANOVA. p-value: *<0.05; **<0.01; ***<0.001; ****<0.0001


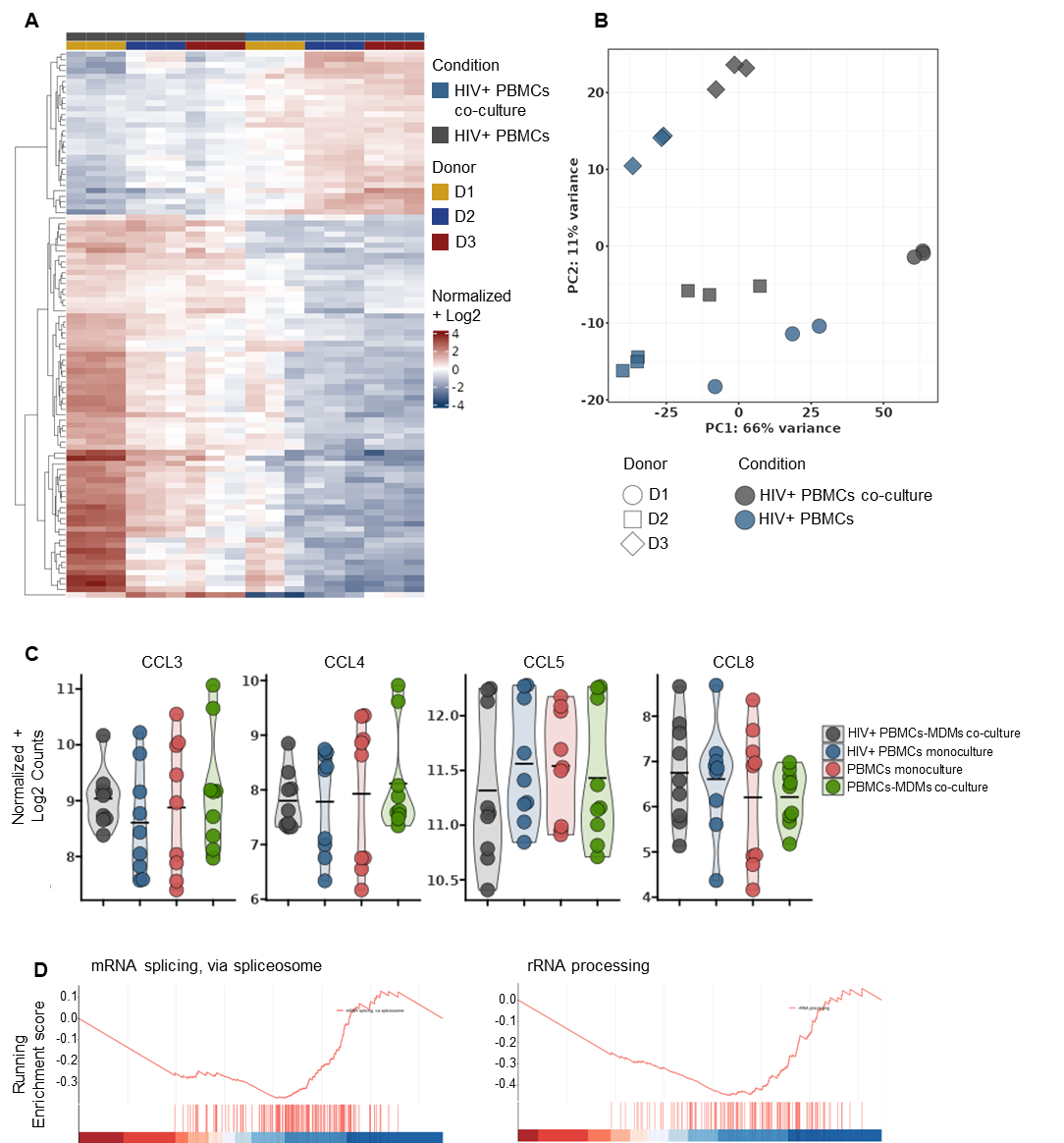


**Fig. S15. Bulk RNA of CD4+ T cells sorted from HIV infected and uninfected PBMCs from monocultures and MDMs co-cultures.** (**A**) Heatmap of top 200 differently regulated genes in HIV infected PBMCs-MDMs cocultures vs HIV infected PBMCs monoculture. (**B**) Principal component analysis (PCA) of HIV infected PBMCs-MDMs cocultures vs HIV infected PBMCs monoculture. (**C**) Violin plots of expression levels of HIV co-receptors ligands in CD4+ T cells in the 4 different culture conditions analysed (mean). (**D**) GSEA showing downregulation of mRNA splicing and rRNA processing in CD4+ T cells of HIV infected PBMCs-MDMs cocultures vs HIV infected PBMCs monoculture. Statistic by DESeq2 (see materials & methods section). p-value: *<0.05; **<0.01; ***<0.001; ****<0.0001.


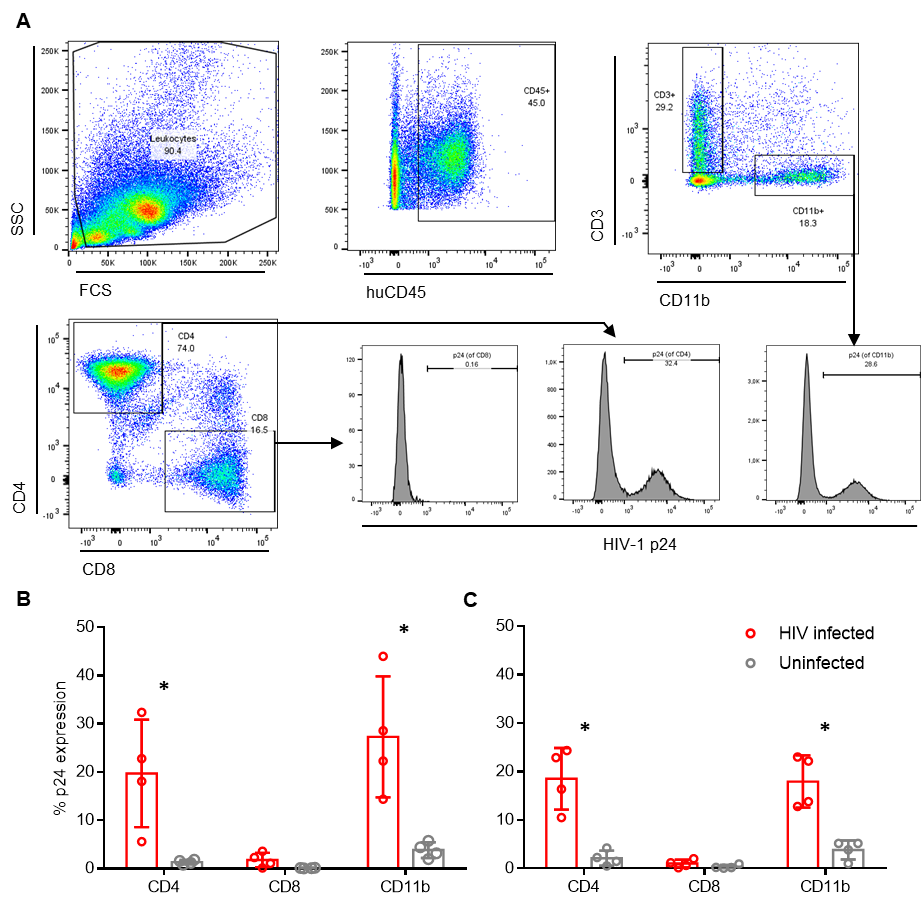


**Fig. S16 HIV p24 detection in human leukocytes in HIV infected hu NSG mice.** (**A**) Gating strategy for evaluation of intracellular HIV p24 expression, evaluated via flow cytometry in CD4 T cells (hCD45+, CD3+, CD11b-, CD4+, CD8-), CD8 T cells (hCD45+, CD3+, CD11b-, CD4-, CD8+) and CD11b monocytes/macrophages (hCD45+, CD3-, CD11b+) of (**B**) bone marrow (**C**) and spleen. Both HIV-1 YU-2 infected (n=4) and uninfected (n=4) mice were evaluated (Mean and standard deviation). Statistics by multiple T test. Mann-Whitney (**B** and **C**). p-value: *<0.05; **<0.01; ***<0.001; ****<0.0001


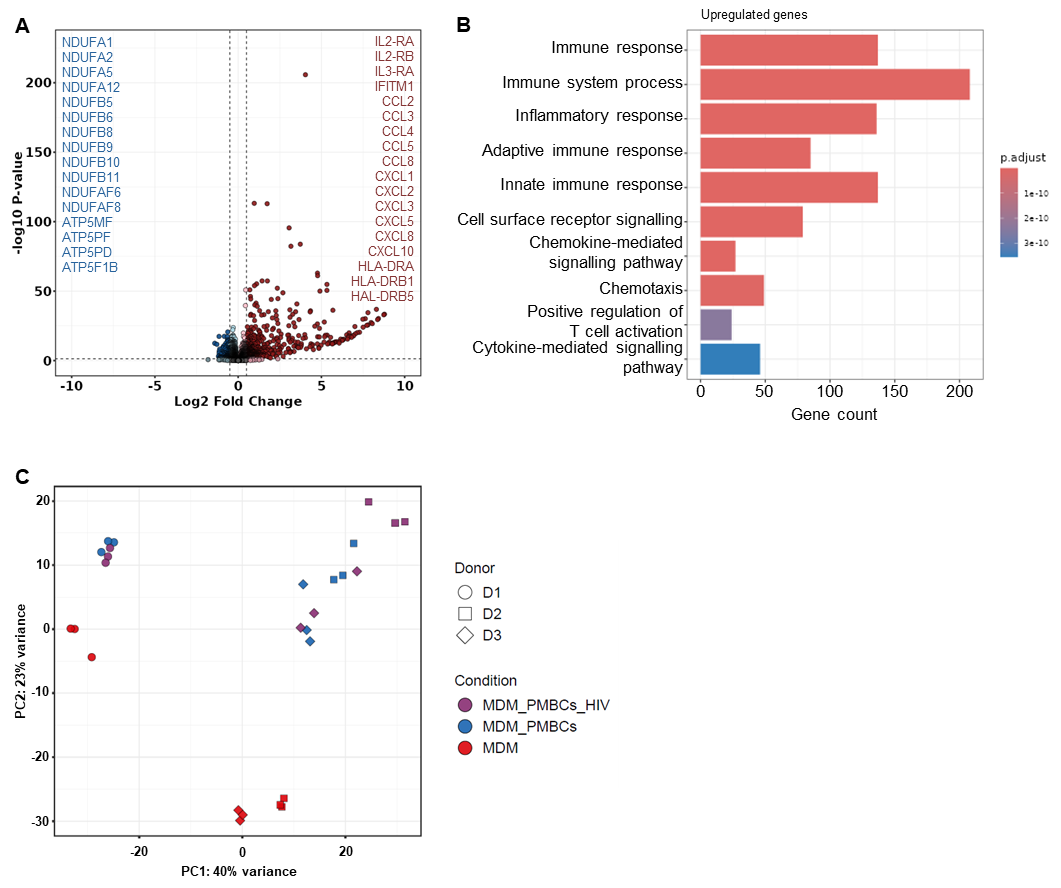


**Fig. S17. Bulk RNA sequencing of pro-inflammatory MDMs, in monocultures or co-cultured with PBMCs.** (**A**)Volcano plot of differently expressed genes in MDMs monocultures vs MDMs-PBMCs co-cultures. Annotated genes with highest significance and fold change. (**B**) ORA of the 10 upregulated biological processes with the highest p-value. (**C**) Principal component analysis (PCA) of RNA from MDM samples. Statistic by DESeq2 (see materials & methods section). p-value: *<0.05; **<0.01; ***<0.001; ****<0.0001.
